# Mitochondrial Mutations in Cholestatic Liver Disease with Biliary Atresia

**DOI:** 10.1038/s41598-017-18958-8

**Published:** 2018-01-17

**Authors:** Hong Koh, Gun-Seok Park, Sun-Mi Shin, Chang Eon Park, Seung Kim, Seok Joo Han, Huy Quang Pham, Jae-Ho Shin, Dong-Woo Lee

**Affiliations:** 1 Department of Pediatrics, Yonsei University College of Medicine, Severance Children’s Hospital, Severance Pediatric Liver Research Group, Seoul, 03722 South Korea; 2 School of Applied Biosciences, Kyungpook National University, Daegu, 41566 South Korea; 3 Department of Pediatric Surgery, Yonsei University College of Medicine, Severance Children’s Hospital, Seoul, 03722 South Korea; 4 Present Address: Department of Biomedical Engineering, University of Texas at Austin, Austin, TX 78712 USA; 5 Present Address: CJ CheilJedang, Food Research Institute, Suwon, 16495 South Korea

## Abstract

Biliary atresia (BA) results in severe bile blockage and is caused by the absence of extrahepatic ducts. Even after successful hepatic portoenterostomy, a considerable number of patients are likely to show progressive deterioration in liver function. Recent studies show that mutations in protein-coding mitochondrial DNA (mtDNA) genes and/or mitochondrial genes in nuclear DNA (nDNA) are associated with hepatocellular dysfunction. This observation led us to investigate whether hepatic dysfunctions in BA is genetically associated with mtDNA mutations. We sequenced the mtDNA protein-coding genes in 14 liver specimens from 14 patients with BA and 5 liver specimens from 5 patients with choledochal cyst using next-generation sequencing. We found 34 common non-synonymous variations in mtDNA protein-coding genes in all patients examined. A systematic 3D structural analysis revealed the presence of several single nucleotide polymorphism-like mutations in critical regions of complexes I to V, that are involved in subunit assembly, proton-pumping activity, and/or supercomplex formation. The parameters of chronic hepatic injury and liver dysfunction in BA patients were also significantly correlated with the extent of hepatic failure, suggesting that the mtDNA mutations may aggravate hepatopathy. Therefore, mitochondrial mutations may underlie the pathological mechanisms associated with BA.

## Introduction

Cholestasis is a condition that leads to bile stasis and the accumulation of potentially toxic bile acids in the liver and the systemic circulation^1^. The condition can be caused by inflammation, viral infection, autoimmune disease, or congenital disorders of the hepatobiliary systems^2–4^. In particular, biliary atresia (BA) is a progressive fibro-inflammatory neonate cholangiopathy that is defined by severe alterations in hepatic morphology and physiology^5–7^. BA is the most common cause of cholestatic liver disease and life-saving liver transplantation in children^8^. Hepatic portoenterostomy (also referred to as the Kasai procedure) is a surgical treatment performed on infants with BA to allow for bile drainage. Although not perfectly curative, this procedure may relieve jaundice and stop liver fibrosis, allowing normal growth and development to occur^9^. After surgery, bilirubin levels will fall to normal levels in about 50% of infants, allowing 40–50% of affected infants to retain their own liver and to reach the age of 5–10 years^10^. The remaining 50% of affected children will not regain adequate bile flow and liver function, and may require a liver transplantation.

Many critical metabolic functions such as gluconeogenesis, triacylglyceride oxidation, fatty acid decomposition, amino acid deamination and transamination, and most plasma protein synthesis are carried out predominantly in hepatocytes^11,12^. Thus, hepatocytes are rich in mitochondria, as each cell contains about ~1,000 mitochondria; ~18% of the entire cell volume. It is clear that impaired mitochondrial function may cause severe damage to hepatocytes, thus affecting many vital cellular functions^13–15^.

Tissue affected by chronic liver disease undergoes persistent and massive apoptosis^16^. Apoptosis-mediated fibrogenesis seems to be tightly associated with alterations in mitochondrial function^17^. Mitochondria play an essential role in the production of adenosine triphosphate (ATP) that is used as a cellular energy currency and reactive oxygen species (ROS) that are involved in signaling, pumping of cytosolic Ca^2+^, and the regulation of apoptosis^18^. Mutations in mitochondrial DNA (mtDNA) can alter the efficiency of cellular energy transduction, resulting in mitochondrial dysfunction^19,20^. Multisystemic disorder results in several distinct syndromes such as the Reye’s syndrome^21^, Wilson’s disease^22^, exercise intolerance^23^, and Zellweger syndrome^24^. Likewise, liver cirrhosis appears to be associated with hepatocytic mitochondrial dysfunction^25^. It remains unclear whether such dysfunctional hepatic mitochondria are transmitted by nonmendelian inheritance, maternal inheritance, or a sporadic condition. Until recently, little is known about the molecular basis of BA, as the sole manifestation of mitochondrial dysfunction in this disease was in sporadic cases. However, several reports regarding biliary atresia splenic malformation syndrome^26^, cystic BA^27^, and cytomegalovirus-IgM(+) associated BA^28^ would suggest that there is a root cause in mitochondrial dysfunction.

**Figure 1:**
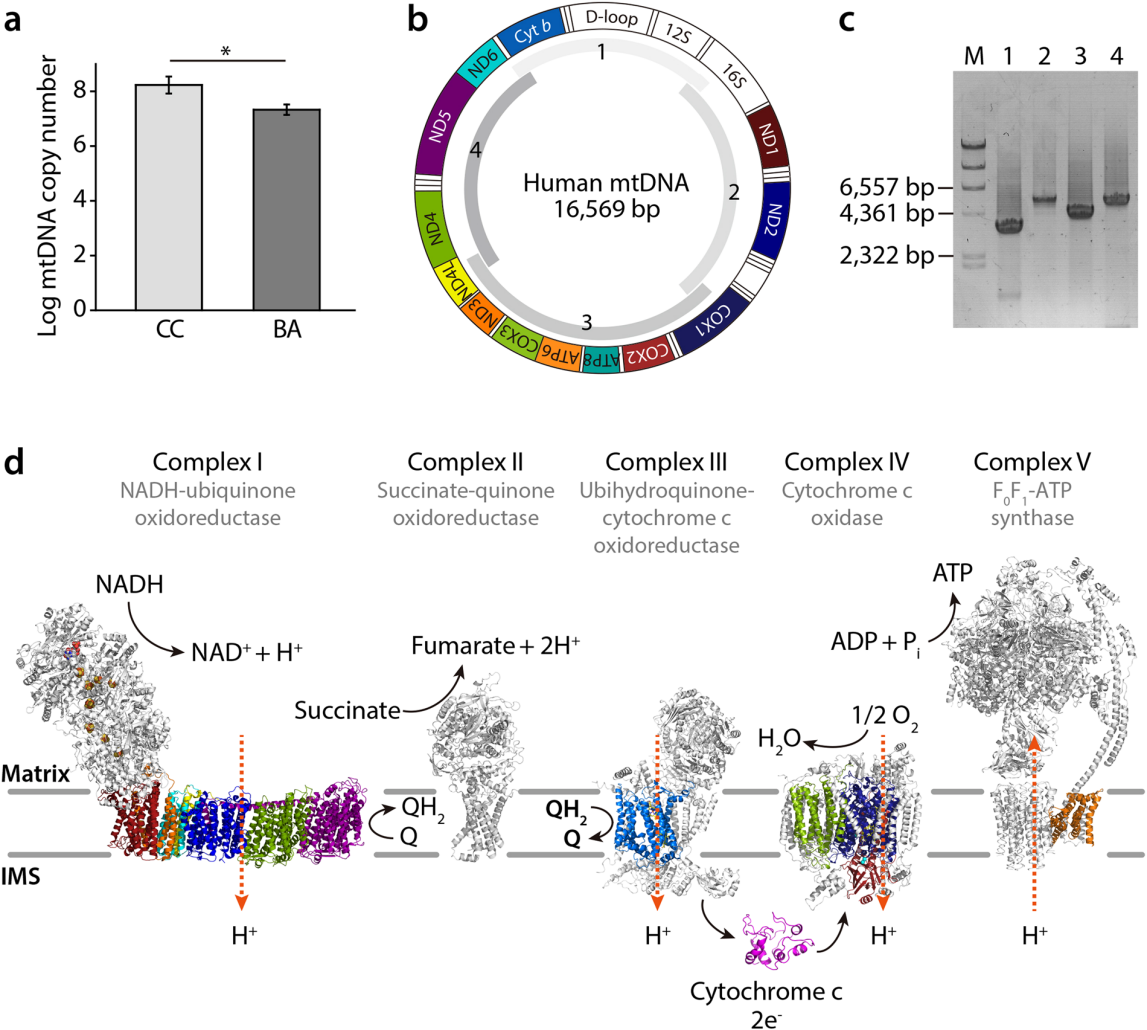
Schematic representation of the human mitochondrial DNA and the mitochondrial respiratory chain. (**a**) Comparison of the mtDNA copy number in plasma between BA and CC patients. The mtDNA copy numbers of CC patient group were slightly higher than those of BA patient group. Data represent the mean ± SE. ∗*p* < 0.05 versus BA. (**b**) The map of the human mitochondrial genome (NC_012920.1) with the protein-coding genes colored according to the complexes to which they contribute subunits, two ribosomal RNAs, 22 tRNAs and non-coding D-loop in white. Montage depicting the structural information currently available for the five complexes that together contribute to the mitocondrial oxidative phosphorylation machinery. (**c**) Electrophoresis of the amplified DNA fragments for mtDNA by PCR. M, *λ/Hin*dIII DNA marker; lane 1, PCR product 1 (3,580 bp); lane 2, PCR product 2 (5,548 bp); lane 3, PCR product 3 (4,447 bp); lane 4, PCR product 4 (5,591 bp). (**d**) The mammalian mitochondrial electron transport chain includes the proton-pumping enzymes complex I (NADH–ubiquinone oxidoreductase), complex III (cytochrome *bc*_1_) and complex IV (cytochrome *c* oxidase), that combined, generate proton motive force that in turn drives F_1_F_O_-ATP synthase. Each complex is embedded in the lipid bilayer with the mitocondrial-encoded subunits colored corresponding to the genome diagram. The structure of each respiratory complex is presented: complex I from *Thermus thermophilus* (protein databank (PDB) code 4HEA), complex II from porcine *Sus scrofa* (PDB 4YXD), complex III from bovine *Bos taurus* (PDB 1L0L), complex IV from bovine *B. taurus* (PDB 1OCC) and complex V from bovine *B. taurus* (PDB 5ARA). The iron-sulfur cofactors of complex I are depicted as orange and yellow spheres. IMS, intermembrane space.

Human mtDNA is found in the mitochondrial matrix and consists of 13 structural genes that encode integral membrane subunits for complexes I, III, IV, and V in the mitochondrial respiratory chain^29^ (Fig. 1). The mitochondrial matrix possesses an incomplete mtDNA repair system, and is highly sensitive to ROS-induced oxidative damage because of its proximity to the inner mitochondrial membrane where most ROS are produced^30–32^. Accumulation of toxic bile acids in the liver, oxidative stress, and systemic circulation negatively affect the mitochondrial function by directly impairing liver respiratory chain activity^33,34^. In addition, non-functional mitochondria are important for the production of ROS, that in turn can promote the onset of apoptosis and are responsible for the activation of profibrogenic mechanisms^35,36^. Major alterations to energy metabolism in experimental cholestasis resemble metabolic alterations that are observed in patients with cirrhosis^37^. Part of the adverse effects of bile acids on mitochondrial bioenergetics could be related to disturbances of mitochondrial membrane composition^33^. Yet, the specific mechanisms involved in the onset and development of human hepatic mitochondrial dysfunction in liver cholestasis remains unclear.

In this study, using a next-generation sequencing technique, we performed a mitochondrial genome-wide analysis to investigate the extent of mtDNA mutations in the liver of 14 BA patients and compare it to that in livers from five choledochal cyst (CC) patients as a control. We particularly focused on the genes encoding energy-transducing components of the respiratory chain. A comparison of clinical, morphologic, biochemical, and genetic features of these patients reveals a remarkable uniformity, suggesting that the mutations in mtDNA protein-coding genes are highly associated with BA without evidence of maternal inheritance.

## Results

### Histological and Biochemical Characteristics of Cholestatic Patients

The clinical characteristics of 19 patients with chronic liver diseases with BA or CC are summarized in Table 1. Between both groups of cholestatic patients, there were distinct characteristics in the parameters of hepatic injury and liver functions. At the time of liver wedge biopsy during surgery, BA patients were relatively 1 year older than CC patients. In addition, their parameters of hepatic injury and liver function such as aspartate aminotransferase (AST), alanine aminotransferase (ALT), AST to Platelet ratio index (APRI), direct bilirubin (D.Bil), gamma-glutamyl transpeptidse (γ-GT), and prothrombine time-international normalized ratio (PT-INR) were statistically significantly higher than those of CC patients (P value < 0.05). These observations indicated that BA patients exhibited more severe damage in liver function than CC patients.

**Table 1:** Comparison of clinical and biochemical characteristics of all liver diseases between BA and CC patients.

	Total	BA	CC	*P*
	(N = 19)	(N = 14)	(N = 5)	
Male (%)	8 (42.1)	7 (50.0)	1 (20.0)	
Age (weeks)	15.88 ± 16.80	19.18 ± 17.73	6.67 ± 10.21	0.033
AST (13.0~34.0 IU/L)*	695.2 ± 2095.5	925.1 ± 2421.4	51.4 ± 21.9	0.002
ALT (5.0~46.0 IU/L)	295.7 ± 620.1	396.0 ± 700.9	15.0 ± 9.4	0.003
T.Bil (0.2~0.8 mg/dL)	8.07 ± 2.73	8.35 ± 1.82	7.28 ± 4.66	NS
D.Bil (0.1~0.4 mg/dL)	4.97 ± 3.06	6.50 ± 1.85	0.70 ± 0.35	0.001
Protein (6.0~8.0 g/dL)	5.75 ± 0.63	5.65 ± 0.70	5.60 ± 0.31	NS
Albumin (3.3~5.3 g/dL)	3.68 ± 0.49	3.66 ± 0.54	3.76 ± 0.36	NS
*γ*-GT (12.0~54.0 IU/L)	578.7 ± 779.7	733.4 ± 860.4	145.8 ± 109.0	0.006
Platelet (150.0~400 10^3^/μL)	354.4 ± 124.4	353.6 ± 133.5	356.8 ± 108.3	NS
PT-INR (0.91~1.16)	1.07 ± 0.31	1.10 ± 0.35	0.98 ± 0.06	0.002
APRI	13.213 ± 48.676	17.771 ± 56.529	0.454 ± 0.245	0.002
METAVIR stage, 0/1/2/3/4	5/0/5/8/1	0/0/5/8/1	5/0/0/0/0	

All data show mean ± SD. AST = aspartate aminotransferase; ALT = alanine aminotransferase; T.Bil = total value bilirubin; D.Bil = direct bilirubin; *γ*-GT = gamma-glutamyl transpeptidase; PT-INR = prothrombine time-international normalized ratio; APRI = AST to platelet ratio index; BA = biliary atresia; CC = choledochal cysts.

To investigate the state of the patients’ livers in detail, we performed liver function tests for all 19 patients with BA or CC (Table 2). All 14 BA patients showed cholestasis with elevated levels of D.Bil and γ-GT together with high levels of AST and ALT that are indicative of hepatic injury. APRI, a non-invasive serologic parameter of hepatic fibrosis, was significantly elevated in ten BA patients (patients 7, 8, 9, 11, 12, 13, 14, 16, 17, and 19). However, we did not find any significant necro-inflammantory changes in histological evaluation. Four of the five CC patients displayed only elevated AST levels, while other clinical characteristics were in the normal range. In these CC patients, liver injury was not significantly correlated with platelet count, protein level, albumin level, and total bilirubin (T.Bil) level. This indicates that CC patients exhibited relatively normal liver function and hepatic structure. Thus, hepatic fibrosis in CC patients did not seem to be directly related to liver mitochondrial dysfunction, whereas BA patients showed severe hepatic damage with high levels of AST and ALT. In particular, histological evaluation demonstrated that nine BA patients (patients 8, 9, 12, 13, 14, 16, 17, 18, and 19) exhibited severe destruction of normal hepatic structure (METAVIR ≥ 3), including hepatic fibrosis and cirrhosis. However, none of the CC patients presented significant hepatic fibrosis, and all displayed a normal hepatic structure (METAVIR = 0). Based on METAVIR and APRI values, significant histological and clinical hepatic fibrosis were observed in eight BA patients (patients 8, 9, 12, 13, 14, 16, 17, and 19). Values for albumin and PT-INR were relatively decreased in four of the eight BA patients (patients 9, 12, 14, and 17), indicating that, in these patients, hepatic synthesis was dysfunctional. However, the other BA patients (patients 8, 13, 16, and 19) appeared to retain hepatic biosynthetic function relatively higher than the former patients (patients 9, 12, 14, and 17) described above. Therefore, our histological and biochemical examination clearly indicated that both CC and BA patients showed liver injury. However, in the case of BA patients, the degree of liver function was different between patients.

**Table 2:** The histological and clinical characteristics of all patients with cholangiopathy.

Patients	Sex	Age (wks)	Dx	METAVIR	AST	ALT	Platelet	APRI	T.Bil	D.Bil	Pro	Alb	γ-GT	PT-INR
1	F	24.9	CC	0	66	31	309	0.628	0.3	0.2	6.0	4.3	24	1.05
2	M	2.9	CC	0	21	9	298	0.207	12.7	1.2	5.6	3.9	307	0.96
3	F	1.9	CC	0	48	12	331	0.427	9.5	0.7	5.3	3.7	73	0.9
4	F	1.9	CC	0	44	8	549	0.236	5.6	0.7	5.3	3.4	142	1.01
5	F	1.7	CC	0	78	15	297	0.772	8.3	0.7	5.8	3.5	183	0.97
6	M	14.9	BA	2	146	162	351	1.223	7.6	6.2	5.6	3.8	307	0.92
7	M	9.0	BA	2	202	98	275	2.160	11.1	9.1	5.4	3.4	382	0.94
8	M	15.4	BA	3	467	362	427	3.217	11.5	9.5	6.3	4.4	406	0.98
9	F	15.3	BA	3	330	244	505	1.922	9.6	7.5	5.2	3.1	644	1.34
10	F	3.9	BA	2	71	11	276	0.757	7.1	4	4.9	3.3	237	0.97
11	M	9.6	BA	2	174	114	379	1.350	6.1	4.9	5.4	3.8	422	0.98
12	F	10.1	BA	3	148	130	197	2.0879	9	7.6	5.0	2.9	1145	1.19
13	M	61.0	BA	3	206	443	391	1.550	4.6	4	7.1	4.2	3371	1.03
14	M	11.3	BA	3	814	134	207	11.566	8.2	6.6	4.5	3.2	475	1.16
15	F	8.6	BA	2	155	179	545	0.837	8.3	6.7	5.6	4	246	0.99
16	F	17.9	BA	3	227	233	463	1.442	8.4	7.1	6.6	4.5	1658	0.85
17	F	58.7	BA	4	9310	2790	128	213.925	8.1	3.5	5.8	3	180	2.23
18	M	12.9	BA	3	184	193	542	0.999	7.6	6.4	6.2	4.2	541	0.81
19	F	19.9	BA	3	517	451	264	5.760	9.7	7.9	5.5	3.4	253	0.99

Age (wks) = age at the time of liver wedge biopsy (weeks); Dx = Diagnosis; AST = aspartate aminotransferase (IU/L); ALT = alanine aminotransferase (IU/L); APRI = AST to platelet ratio index; T.Bil = total bilirubin (mg/dL); D.Bil = direct bilirubin (mg/dL); Pro = Protein (g/dL); Alb = Albumin (g/dL); γ-GT = gamma-glutamyl transpeptidase (IU/L); PT-INR = prothrombine time-international normalized ratio.

### Comparison of total DNA extracted by direct sequencing and mtDNA amplicon sequencing

To compare the copy numbers of mtDNAs between the BA and CC patients, we calculated the relative copy number (Rc) = 2 ΔCt (Ct_β-actin_ − Ct_mtDNA_). As shown in Fig. 1a, BA patients exhibited a slightly lower mtDNA copy numbers than CC patients. Next, in order to examine whether conventional PCR-based amplicon sequencing provides additional nucleotide variation in hepatic mtDNAs due to PCR bias, we performed direct and amplicon sequencing with the same patient samples. For this, we chose three BA patients (Patients 12, 17, and 18) out of 14 BA patients. These three patients exhibited different clinical features with respect to the extent of liver injury and liver function as described above (Tables 1 and 2). For direct sequencing, we isolated mitochondria from liver tissues, extracted total DNA, and constructed a sequencing library. In the case of amplicon sequencing, mtDNA was amplified by PCR using four pairs of overlapping primers, yielding 3,580 bp, 5,548 bp, 4,447 bp, and 5,591 bp DNA fragments (Fig. 1b and c). The amplified PCR products were also used to prepare the mtDNA sequencing library. An Ion Xpress Barcode Adapter was used to separate sequence data belonging to individual patients for both direct sequencing libraries and mtDNA amplicon sequencing prior to emulsion PCR. Both libraries were then subjected to the Ion Torrent Personal Genome Machine (PGM) sequencer system. As described in Table 3, our results showed a relatively low coverage (<50×) with a <1% mapped mtDNA read percentage, mainly due to cross-contamination with nuclear DNA (nDNA) as expected. On the other hand, mtDNA amplicon sequencing exhibited a high coverage (>2,000×) with a >92% mapped mtDNA read percentage. The vastly different metrics generated by the two sequencing approaches did not give any discrepant or biased results between them, indicating that mtDNA amplicon sequencing could provide similar results to direct sequencing with respect to the total number of identified single nucleotide variations (SNV). Indeed, we found almost the same number and location of SNVs for Patient 12 from the direct sequencing and mtDNA amplicon sequencing results. Remarkably, mtDNA amplicon sequencing found a 14766 C > T variation in Patient 17, and 16183 A > C and 16189 T > C variations in Patient 18, that were not detected by direct sequencing because of insufficient mapped reads ascribed to its low average coverage (<40×) (Table 3 and Table S2). On the other hand, a 2156 A insertion in Patient 12, a 523–524 deletion and a 2156 insertion A in Patient 17, and a 281–8289 deletion in Patient 18 were not detected by amplicon sequencing mostly because of device-specific indel errors (Table 3 and Table S1). Overall, these results indicate that mtDNA amplicon library sequencing is a more efficient approach than direct sequencing to verify the mtDNA sequences of liver tissues.

**Table 3:** Sequencing metrics of total DNA extract and mtDNA amplicon.

Sample	12		17		18	
	Total DNA extract	mtDNA amplicon	Total DNA extract	mtDNA amplicon	Total DNA extract	mtDNA amplicon
Total reference length (bp)	16,569	16,569	16,569	16,569	16,569	16,569
Total read count	521,196	226,135	499,861	314,756	511,511	251,829
Mapped mtDNA read count	4,899	210,745	2,482	293,863	3,093	232,972
Mapped read percentage (%)	0.94	93.19	0.50	99.36	0.60	92.51
Average coverage	42×	2,034×	22×	2,705×	26×	2,164×
SNV row count	33	32	33	32	25	26

Runs of all three patients’ samples were performed independently in triplicates. mtDNA = mitochondrial DNA; SNV = single nucleotide variation.

### Molecular Genetic Analysis for Cholestatic Patients

To rapidly detect SNVs, mtDNA amplicon libraries derived from mtDNAs for 19 cholestatic patients were constructed. We obtained a total of 4,734,991 raw reads for all 19 samples with an average coverage of >1,000× (Table S2). From 19 sets of mtDNA sequences, 678 SNVs were identified. Out of these SNVs, 151 non-synonymous variations were found in the 13 mitochondrial protein coding sequences. As described in Table 4 and Table S3, the CYB region had the highest number of non-synonymous SNVs (41 SNVs), followed by 39 SNVs in ATP6, 17 SNVs in DN4, 16 SNVs in ND2, 10 SNVs in ND3, 7 SNVs in COX2, 6 SNVs in ATP8, 6 SNVs in ND1, 5 SNVs in ND5, 3 SNVs in COX1, and one SNV in COX3, respectively. SNVs were not identified in ND4L and ND6. Remarkably, novel SNVs in BA patients were identified in COX2 (L179 [X, stop]) in six samples (patients 6, 7, 11, 12, 17, and 18), ATP8 (M42X) in one sample (patient 1), ND2 (T119A) in 4 samples (patients 9, 12, 14, and 17), and ND4 (S97X) in eight samples (patients 1, 5, 8, 13, 14, 15, 16, and 19) in the study. Novel SNVs had relatively low frequency (35.64~69.32%) when compared with the frequency of other SNVs (Table S3). All samples, except the reference human mitochondrion genome (http://www.ncbi.nlm.nih.gov/nuccore/251831106), showed a haplogroup that originated in Asia (Table S2).

**Table 4:** Non-synonymous variations in mtDNA encoding regions.

Patient		Locus										
		Complex I					Complex III	Complex IV			Complex V	
Type	No.	*ND1*	*ND2*	*ND3*	*ND4*	*ND5*	*Cyt b*	*COX1*	*COX2*	*COX3*	*ATP6*	*ATP8*
CC	1		L237M (C5178A)	T114A (A10398G, C10400T)	Y95H(T11042C)/S97X (T11048del)		T7I(C14766T)/I78T(T14949C)/T194A (A15326G)				T59A(A8701G)/T112A (A8860G)	L17F(C8414T)/ M42X (T8473del)
	2					T8A (A12358G)	T7I(C14766T)/I115T(T15090C)/T194A (A15326G)				T112A (A8860G)	
	3	T275A* (A4129G)				T8A (A12358G)	T7I(C14766T)/T194A (A15326G)				T112A (A8860G)	
	4		T122A (A4833G)	T114A (A10398G, C10400T)			T7I(C14766T)/T194A (A15326G)				T59A(A8701G)/T112A (A8860G)	
	5		L237M (C5178A)	T114A (A10398G, C10400T)	Y95H(T11042C)/S97X (T11048del)		T7I(C14766T)/T194A (A15326G)	V128I (G6285A)			T59A(A8701G)/T112A (A8860G)	L17F (C8414T)
BA	6		L237M (C5178A) /I278V (A5301G)	T114A (A10398G, C10400T)			T7I(C14766T)/T194A (A15326G)	M117T (T6253C)	L179X (T8119del)		T59A(A8701G)/T112A (A8860G)	
	7		L237M(C5178A)/I278V (A5301G)	T114A (A10398G, C10400T)	I423V (A12026G)		T7I(C14766T)/T194A (A15326G)		L179X (T8119del)		T59A(A8701G)/T112A (A8860G)	
	8	V113A (T3644C)	L237M (C5178A)	T114A (A10398G, C10400T)	Y95H(T11042C)/S97X (T11048del)		T7I(C14766T)/T194A (A15326G)				T59A(A8701G)/T112A (A8860G)	L17F (C8414T)
	9		T119A (A4824G)			F182L (T12880C)	T7I(C14766T)/T194A (A15326G)				T13A(A8563G)/H90Y(C8794T)/T112A (A8860G)	
	10		L237M (C5178A)	T114A (A10398G, C10400T)		T449A (A13681G)	T7I(C14766T)/T194A (A15326G)				T59A(A8701G)/T112A (A8860G)	L17F (C8414T)
	11						T7I(C14766T)/T194A (A15326G)		L179X (T8119del)		T112A(A8860G)/V142I(G8950A)	
	12		T119A (A4824G)				M4T(T14757C)/T7I(C14766T)/T194A ( A15326G)	V338M (G6914A)	L179X (T8119del)		T13A(A8563G)/H90Y(C8794T)/T112A (A8860G)	
	13	T153M (C3764T)	L237M (C5178A)	T114A (A10398G, C10400T)	Y95H(T11042C)/S97X (T11048del)		T7I(C14766T)/T194A (A15326G)				T59A(A8701G)/T112A (A8860G)	L17F (C8414T)
	14		T119A (A4824G)		Y95H(T11042C)/S97X (T11048del)		T7I(C14766T)/T194A (A15326G)				H90Y(C8794T)/T112A (A8860G)	
	15			T114A (A10398G, C10400T)	Y95H(T11042C)/S97X (T11048del)		T7I(C14766T)/T194A (A15326G)				A20T/T59A(A8701G)/T112A (A8860G)	
	16	Y43C(A3434G)/A64V (C3497T)			Y95H(T11042C)/S97X (T11048del)		T7I(C14766T)/T194A (A15326G)				T112A (A8860G)	
	17		T119A (A4824G)			F182L (T12880C)	T7I (C14766T)		L179X (T8119del)		T13A(A8563G)/H90Y(C8794T)/T112A (A8860G)	
	18	A64V (C3497T)					T7I(C14766T)/T194A (A15326G)		T63A(A7772G)/ L179X (T8119del)		T112A (A8860G)	
	19		L237M (C5178A)/I278V (A5301G)	T114A (A10398G, C10400T)	Y95H(T11042C)/S97X (T11048del)		T7I(C14766T)/T194A (A15326G)			V248I (G9948A)	T59A(A8701G)/T112A(A8860G)/A207T (G9145A)	

*Human residue (Nucleotide)

Runs of all 19 patients‘ samples were performed independently in triplicates.

### Structural Analysis of mtDNA Mutations in Respiratory chains

**Figure 2:**
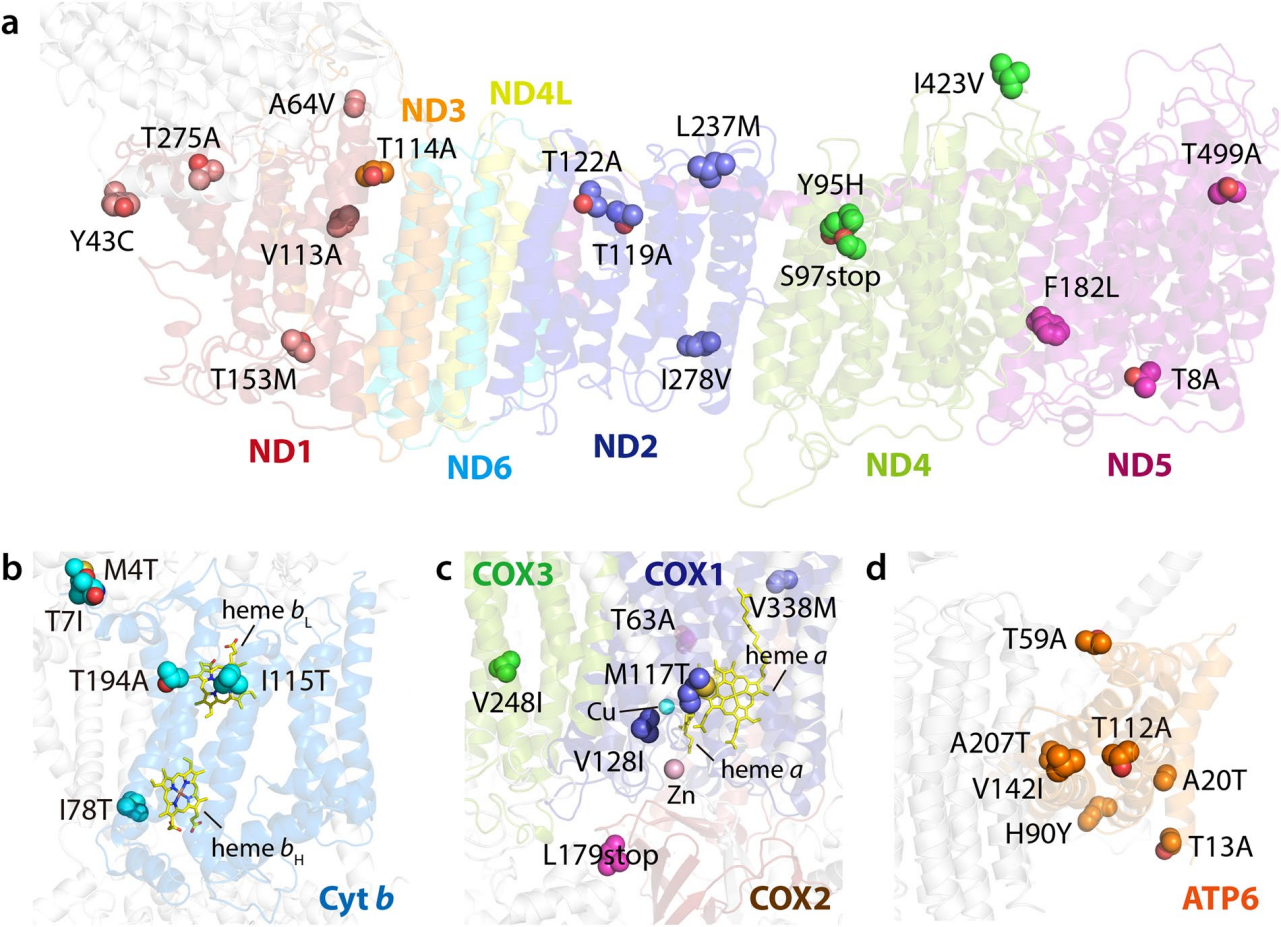
Location of putative mitochondrial mutation sites associated with BA cholestasis in complexes I to V. Close-up views of the ND subunits of complex I (**a**), Cyt *b* of complex III (**b**), three Cox monomers of complex IV (**c**), and ATP6 of complex V (**d**). Thirty-four individual mutation sites are mapped onto their corresponding 3D model structures depicted as transparent ribbon representation. The side chain of each mutation site is shown as a sphere colored by atom type (oxygen in red and nitrogen in blue, respectively) with the mitochondrial-encoded subunits. The heme cofactors of Cyt *b* and COX2 subunits are depicted as yellow stick representations, and Cu and Zn cofactors of COX2 depicted as spheres colored in cyan and pink, respectively. More detailed locations of mutation sites can be found in Figures S1 to S11.

We aimed to understand the potential effect of mtDNA mutations on the functionality of the respiratory chain. For this, we investigated whether the mutational sites of mtDNA protein-coding genes were structurally associated with their assembly factors, causing mitochondrial dysfunction. We constructed 3D model structures of human mitochondrial complexes based on the most homologous protein structures available (i.e., *Thermus aquaticus thermophilus* for complex I and *Bos taurus* for complexes III, IV, and V) using the SWISS-MODEL program (Figs 1d and 2). Additionally, we analyzed the predictive effects of mutations on the structure and function of respiratory chain complexes using SIFT (http://sift.jcvi.org), PROVEAN (http://provean.jcvi.org), and Polyphen-2 (http://genetics.bwh.harvard.edu/pph/) web servers (Table 5). These amino acid substitutions (AAS) algorithms were combined to investigate the effect of mutation on the biological functions of proteins, with an improved prediction accuracy of >69% when analyzed with SNPs known to be associated with mitochondrial diseases^38,39^. Notably, 34 frequent non-synonymous variations found in mtDNA protein-coding genes for both BA and CC patients were not identical to SNPs known for any disease-associations reported in the MITOMAP database. Subsequently, the mutations found in this study were mapped onto their human model structures (Figs 2 and S1 to S11).

**Table 5:** Structural classification and pathogenic prediction of mutations in the mtDNA protein-coding genes.

Human subunit (Gene)	Human residue (Nucleotide)	Predictions					
		SIFT Score^*^	Prediction	PROVEAN Score^§^	Prediction (Cutoff = −2.5)	Polyphen Score^£^	Prediction
ND1	Y43C	0.02	affected	−6.159	deleterious	0.004 (sensitivity: 0.97; specificity: 0.59)	benign
	A64V	0.51	tolerated	−1.405	neutral	0.002 (0.99; 0.30)	benign
	V113A	0.00	affected	−3.333	deleterious	0.052 (0.94; 0.83)	benign
	T153M	0.12	tolerated	0.284	neutral	0.458 (0.89; 0.90)	damaging
	T275A	1.00	tolerated	2.989	neutral	0.001 (0.99; 0.15)	benign
ND2	T119A	0.16	tolerated	−4.241	deleterious	0.771 (0.85; 0.92)	damaging
	T122A	0.01	deleterious	−0.955	neutral	0.665 (0.86; 0.91)	damaging
	L237M	0.35	tolerated	0.391	neutral	0.992 (0.70; 0.97)	damaging
	I278V	0.29	tolerated	−0.513	neutral	0.005 (0.97; 0.74)	benign
ND3	T114A	0.66	tolerated	−1.393	neutral	0.000 (1.00; 0.00)	benign
ND4	Y95H	0.00	deleterious	−3.729	deleterious	0.999 (0.14; 0.99)	damaging
	S97X			−9.238	deleterious		
ND5	T8A	0.38	tolerated	−1.344	neutral	(score is not available)	unknown
	F182L	1.00	tolerated	−4.330	deleterious	0.993 (0.70; 0.97)	damaging
	T449A	0.52	tolerated	−0.253	neutral	0.006 (0.97; 0.75)	benign
Cyt *b*	M4T	0.11	tolerated	−1.128	neutral	0.000 (1.00; 0.00)	benign
	T7I	0.01	deleterious	−2.354	neutral	0.001 (0.99; 0.15)	benign
	I78T	0.02	deleterious	−2.500	deleterious	0.661 (0.86; 0.91)	damaging
	T194A	0.35	tolerated	0.312	neutral	0.000 (1.00; 0.00)	benign
COX1	M117T	0.21	tolerated	−2.077	neutral	0.000 (1.00; 0.00)	benign
	V128I	0.16	tolerated	−0.464	neutral	0.240 (0.91; 0.88)	benign
	V338M	1.00	tolerated	1.043	neutral	0.000 (1.00; 0.00)	benign
COX2	T63A	0.07	tolerated	−2.121	neutral	0.017 (0.95; 0.80)	benign
	L179X			−13.674	deleterious		
COX3	V248I	0.12	tolerated	−0.842	neutral	0.000 (1.00; 0.00)	benign
ATP6	T13A	0.22	tolerated	−2.726	deleterious	0.994 (0.69; 0.97)	damaging
	A20T	0.21	tolerated	−0.404	neutral	0.004 (0.97; 0.59)	benign
	T59A	0.66	tolerated	−0.935	neutral	0.002 (0.99; 0.30)	benign
	H90Y	1.00	tolerated	0.238	neutral	0.002 (0.99; 0.30)	benign
	T112A	0.21	tolerated	−3.967	deleterious	0.000 (1.00; 0.00)	benign
	V142I	1.00	tolerated	0.118	neutral	0.000 (1.00; 0.00)	benign
	A207T	0.00	deleterious	−3.543	deleterious	0.999 (0.14; 0.99)	damaging
ATP8	L17F	0.29	tolerated	−1.984	neutral	0.994 (0.69; 0.97)	damaging
	M42X			−7.636	deleterious		

^*^Ranges from 0 to 1. The amino acid substitution is predicted damaging if the assigned SIFT score is <= 0.05, and the substitution is tolerated if the SIFT score is >0.05.

^§^Variants with a score equal to or below−2.5 are considered “deleterious” and variants with a score above −2.5 are considered “neutral”.

^£^The conservation of a position in the multi-sequence alignments and the deleterious effect on the protein structure results in the Position-Specific Independent Count (PSIC) score that ranges from 0 to 1. The classification of the nsSNPs results in Possibly Damaging and Probably Damaging (PSIC > 0.5) or Benign (PSIC < 0.5). mtDNA = mitochondrial DNA.

Out of 45 different polypeptide subunits in human complex I, seven ND subunits that are encoded by mtDNA play an important role in the correct assembly of complex I and the ability to reduce oxygen with formation of superoxide anion^40^. Moreover, they are also critical for supercomplex formation of OXPHOS system^41^. As shown in Fig. 2, several mutations found in ND 1 through ND5 subunits, except ND4L and ND6 subunits, occur in critical locations near the single electron escaping sites such as FMN, Fe-S clusters and ubisemiquinone binding site and intersubunit-contact regions. In particular, T275A (ND1), L237M (ND2), T119A (ND2), T114A (ND3), Y95H (ND4), and T8A (ND5) seem to be critical presumably because these mutations are located in the intersubunit-contact regions (i.e., ND3-ND4L^42^ and ND4-ND5^43^) for the activity and assembly of whole complex I. Among them, T119A and Y95H were assigned as deleterious by AAS prediction (Table 5). In addition, truncation by S97X mutation in ND4 seems to severely affect the correct assembly of complex I. In addition, L237M (ND2) and I278V (ND2) may cause the electron leak because these sites are proximal to the N2 cluster of the neighboring NuoB (NQO6/PSST) subunit^44^.

In case of cytochrome *b* in complex III, I115T and T194A are located near the heme *b*_L_ site responsible for quinone/semiquinone reduction. I78T is also proximal to the heme *b*_H_ site for quinol oxidation. Thus, these mutations may reduce the electron transferring activity of complex III including proton translocation. However, these mutations were not assigned as deleterious based on our AAS models.

For complex IV, M117T and V128I near the nuclear Cu center and di-hemes may hinder the reduction of oxygen to H_2_O. Since COX2 is centered between COX1 and COX3 subunits to assemble the transmembrane subunits, impaired COX2 subunit caused by a L179X mutation is deleterious to the correct assembly of complex IV, which was supported by our AAS prediction analyses. Subunits ATP6 (or subunit *a*) and ATP8 (or A6L) functioning as stators contribute to the stabilization of holocomplex V and to monomer-monomer interactions in mammalian mitochondria^45,46^. Notably, there are six mutations found in ATP6 where rotation is generated by the translocation of protons through the interface between the C8-ring and ATP6. Based on the fact that the γ, δ, and e subunits in the F_1_ catalytic domain are bound to the C8-ring, and together these subunits constitute the rotor^47^, it is likely that these mutations affect the catalytic activity of complex V presumably because of perturbation of correct assembly. However, no mutation was found in subunit ATP8 (A6L) that helps to keep subunit ATP6 in contact with the rotating C8-ring.

Overall, we found 34 SNVs in CC and BA patient mtDNA. Several mutations seem to be critical for mitochondrial functionality, as the corresponding residues are located in the critical region of correct assembly and/or intersubunit contacting region for biological function of the respiratory chain (Fig. 2). Together with AAS model-based bioinformatic analyses and clinical features, we assessed the potential impact of these mutations on mitochondrial dysfunction in cholangiopathic patients (Table 5). We concluded that some mutations found in this study might be associated with mitochondrial dysfunction, presumably underlying the pathogenicity of novel mutation in mtDNA protein-coding genes specific for cholestasis.

## Discussion

Patients with chronic liver diseases caused by BA, which is an important cause of liver failure at infancy and childhood, undergo progressive hepatological deterioration^48^. BA is still the most common cause of liver transplantation in children, even after successful Kasai operation. Cholestasis after surgery can lead to the end-stage liver disease, caused by mitochondrial damage in hepatocytes. Until now, the pathophysiology of the condition was unknown. However, it is known that most chronic liver diseases have defects of mitochondrial energy production, such as deficiency of an enzyme of the mitochondrial respiratory chain complex^20,24^. For example, recent studies show that mutations in protein-coding mtDNA genes and/or mitochondrial genes in nDNA are associated with hepatocellular dysfunction^49–51^. Therefore, the coding region of mtDNA in hepatocytes may play a role in the generation of cholestasis. However, no studies have previously thoroughly reported on the combined hepatopathic effects of variants in the hepatic mtDNA. Indeed, abnormal mitochondrial function was present in a cirrhosis cohort comprising 45 patients with liver failure^50^. In addition, Zeharia and coworkers reported that two patients suffered from a lethal infantile neurodegenerative disorder accompanied by hepatocellular dysfunction^52^. Based on increased plasma lactate and alanine levels, and an abnormal urinary organic acid profile with 3-methylglutaconic aciduria and excessive excretion of Krebs cycle metabolites, mitochondrial respiratory chain defect was suspected. This observation was supported by decreased activity in the mitochondrial respiratory chain complexes I, III, and IV in the patients’ livers using exom analysis. In addition, an established mouse model demonstrated that the distinct metabolic alterations in mice with a mitochondrial polymorphism associated hepatic mitochondrial dysfunction was linked to a non-synonymous gene variation (nt7778 G/T) of ATP8^53^.

BA hepatopathy usually appears because of mutations in one or more of the 11 genes encoding subunits of complexes I, III, IV, and V (Table 4). In this study, we determined the sequences of the protein-coding mtDNA genes in 14 subjects with BA and in five control subjects with CC. In both CC and BA patients, three mutations in mtDNA were found dominantly in ATPase 6 of complex V (i.e., T112A) and cytochrome *b* of complex III (T7I, and T194A), which suggests that these mutations are highly associated with liver dysfunction. Additional frequent mutations (L237M, T114A, and L17F) were also found in ND2 and ND3 of complex I and ATP8 of complex V, respectively. All 14 BA patients had hepatitis that began at infancy and worsened progressively (Table 1). For reasons that are not clear, mitochondrial proliferation is not prominent in these two patient groups.

Consequently, NGS-based genomic studies showed that mutations in complex I in 12 BA patients, although all 14 BA patients exhibit genetic heterogeneity in this condition. Furthermore, all 8 patients who have the of mutation of mtDNA in ND2 and ND4 of complex I showed more severe hepatic dysfunction such as METAVIR, APRI, AST, ALT, and PT-INR rather than other BA and CC patients (Table 1). Notably, four (i.e., 9, 12, 14, and 17) of the 14 BA patients displayed distinct pathogenic mutations (T119A and I278V) in another mtDNA protein-coding gene, ND2. Indeed, hepatic malfunction and liver impairment were severely present in all four of these patients (Table 2). Another set of mutations in ND4 of complex I (i.e., Y95H and S97X) was found in four (i.e., 8, 13, 16, and 19) BA patients. Those patients’ livers were severely fibroticly (fibrotic-Ly) damaged, with relatively preserved hepatic functionality. Liver biopsies of these patients showed septal fibrosis, cholestasis, portal and lobular lymphocytic infiltration with cirrhosis. The mutations were considered pathogenic on the basis of several findings. First, all of the mutations were heteroplasmic, which is associated with deleterious mutations rather than neutral polymorphisms. Y95H and S97X were non-synonymous mutations, resulting in truncated ND4 molecules. The other two mutations (T119A and I278V) for ND2 were substitutions of highly conserved nucleotides.

**Figure 3:**
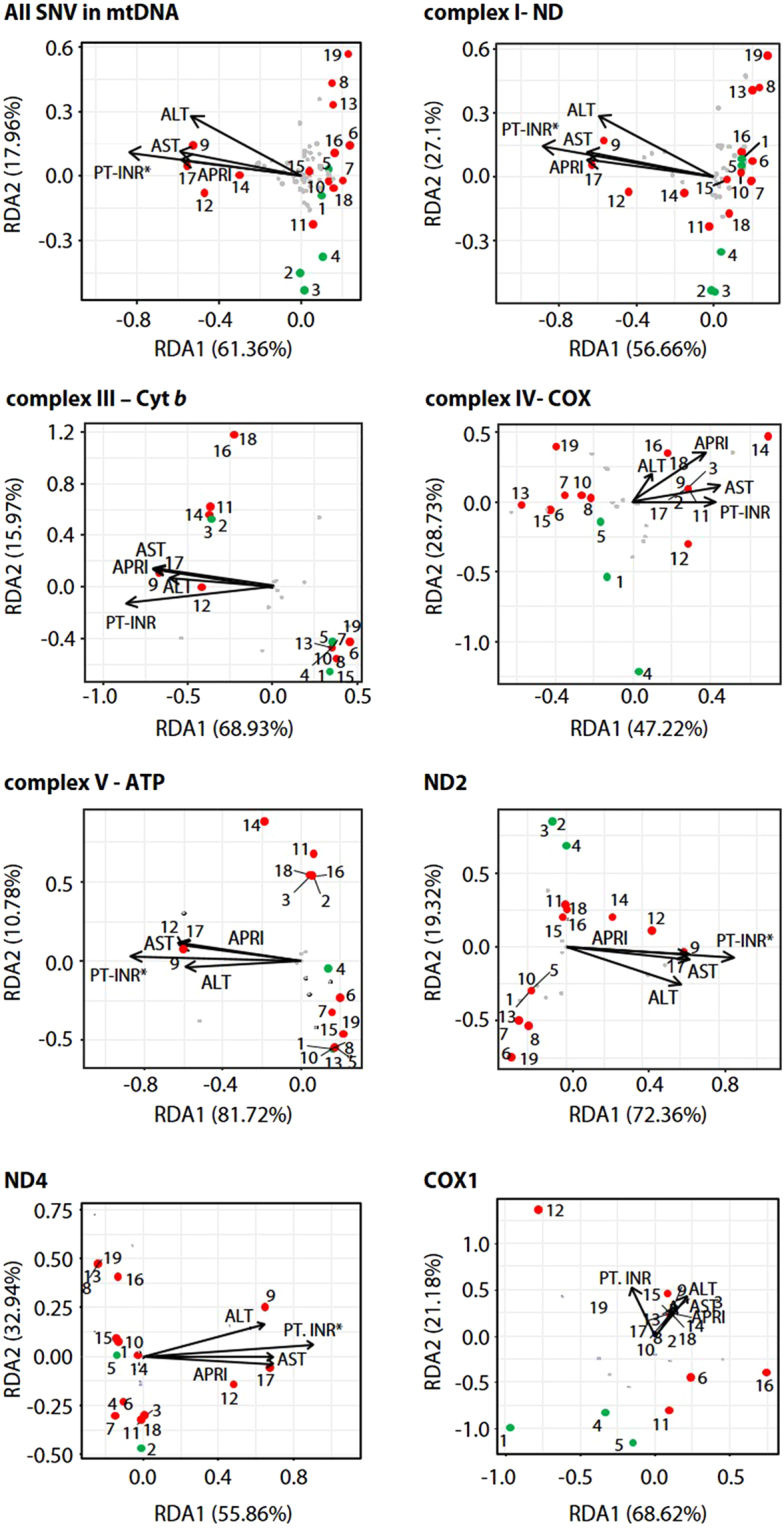
Redundancy analysis triplot of 14 BA and 5 CC patients in their genetic variations and relevant clinical factors. BA (red circles) and CC (green circles) patients; *, statistically significant predictors of the phenotype distribution (anova.cca permutation test, *P* < 0.1).

In all BA patients having the mutations described above the clinical and biochemical characteristics of their liver functions showed a strong correlation between pathologic changes (cirrhosis) and the frequency of mutant mtDNA. It is noteworthy that mtDNA copy number (mtCN) in BA patients was slightly lower than those in the CC patients (Fig. 1a**)**, indicating that BA patients contained to some extent defective mitochondria that affect subsequent cell growth and morphology^54^. In particular, statistical analysis of heteroplasmic sites in mtDNA protein-coding genes between BA (i.e., 9, 12, 14, and 17) and CC patients clearly shows that clinical values of PT-INR, AST, ALT, and APRI significantly positively correlated with hepatic dysfunction (Fig. 3). Consequently, statistical analysis of heteroplasmies clearly support the notion that the parameters of chronic liver injury (i.e., METAVIR, PT-INR, APRI, AST, and ALT) positively correleated with the extent of hepatic failure due to critical mutations in mtDNA protein-coding genes (i.e., ND2 and ND4) in BA patients (i.e., 8, 9, 12, 14, 16, 17, and 19) when compared to CC patients. Recently, it has been reported that skeletal muscle samples exhibit an age-related decrease in mtCN, while there was an age-related increase in mtCN for liver samples^55^. Although heteroplasmies at mutational sites in ND2 and ND4 are highly specific for BA, such mutations did not seem to be accumulated with aging due to the fact that one BA group (i.e., 9, 12, 14, and 17) with more severe hepatic dysfunction is relatively younger than the other BA group (i.e., 8, 13, 16, and 19) (Table 2).

Mutations in the ND4 protein are very critical for Leber Hereditary Optic Neuropathy; a disorder associated with oxidative phosphorylation deficiency^56,57^. Recent studies suggest that mutated ND2 protein impairs mitochondrial complex I assembly, which leads to Leigh syndrome^58^ and exhibits phenotypes that resemble symptoms of mitochondrial disease due to deficient proton pumping activity^59^. Both ND4 and ND2 subunits of complex I are located in proximity to the other mitochondrial subunits in mitochondrial membranes, and ND2 must be functional for proton transfer to occur. All of the non-synonymous mutations are located within or close to the contact region between intersubunits and are likely to prevent assembly and proton pumping pathway, with loss of enzyme activity (Fig. 2). From this perspective, we strongly believe that the mutations found in BA patients are unique and pathogenic when compared to mutation sites found in other condition for hepatic dysfunction^49–51^; however, a functional assay remains to be conducted in cultured cells expressing these genetic mutations. The high frequency of somatic mutations in the cytochrome *b* and ATPase 6 coding genes may seem surprising, however a large number of polymorphisms were observed in the same gene in CC patients (Table 4). Moreover, the fact that all pathogenic mutations identified in our BA patients consisted adenine to guanine substitutions further speculates that these mutations may result from oxidative damage (Table S3). At this stage, we cannot conclude that the mutations in the ND2 and ND4 genes of complex I in our BA patients are definitely somatic. The finding of hepatopathy in a patient with BA and no family history of hepatic disorder should alert the clinician to the possibility of either a ND2 or ND4 gene mutation. Confirmation of the diagnosis requires liver biopsy to investigate complex I deficiency and to identify the molecular defect.

Further study to identify the exact mechanism of hepatic mitochondrial damage in chronic liver disease is urgently required to understand the progression to the end-stage liver disease. Despite the numerous mitochondrial pathways causing mitochondrial diseases, we confined our analysis to mtDNA mutations in the respiratory chain. Nevertheless, mtDNA analysis on BA patients using an NGS-based amplicon library sequencing revealed that all mtDNA samples derived from 15 mg liver tissues were sequenced deeply enough for accurate SNV detection with average coverage of over 1000× and the mapped read percentage of over 92%. Thus, we anticipate that the amplicon sequencing enables us to identify SNPs in hepatic mtDNAs. Consequently, we found that mutations in ND2 and ND4 seem to be highly correlated with hepatic dysfunction particularly in BA patients. This may be a promising approach when integrated with current useful bioinformatic tools to establish the mitochondrial pathophysiology and biochemical mechanism of hepatic dysfunction in chronic liver disease. In fact, those mutational effects remains challenging because of the complex architecture of the respiratory chain, supramolecular arrangement^60^, and significant cross-talk with nuclear-encoded proteins^61^. Nevertheless, mtDNA coding region variants in this study are suggested as potential genetic risk factors for the generation of cholestasis conditions such as BA.

## Materials and Methods

### Patients characteristics

Nineteen pediatric patients with cholangiopathy (1.9~61.0 week-old children with BA and CC) were enrolled at Severance Children’s Hospital (Yonsei University, Seoul, Korea) between August, 2011 and July, 2014. This study was approved by Severance Hospital Institutional Review Board (No. 4-2010-0435), and study protocol was conducted in accordance with the Declaration of Helsinki. Patients were enrolled in this study after written informed consent including the use of liver tissue was obtained from each patient. As a control group, the CC group was defined as children that exhibited no abnormal liver function, showed normal liver histology and only had a cystic dilatation of bile duct. For all pediatric patients, the demographic and biochemical parameters such as sex, age in weeks, serum aspartate aminotransferase (AST), alanine aminotransferase (ALT), total bilirubin (T.Bil), direct bilirubin (D.Bil), protein, albumin, and gamma glutamyl transpeptidase (*γ*-GT), were included at the time of liver wedge biopsy during surgery.

### Collection and qualification of liver tissue

Liver wedge biopsy specimens were collected from the patients at surgery (Kasai hepatoportoenterostomy or liver transplantation in BA group, choledochojejunostomy with a Roux-en-Y anastomosis of dilated bile duct in CC group). All liver tissues were immediately frozen in liquid nitrogen and stored at −70 °C until used. For qualification of liver tissues of BA as a disease group and CC as a control group, liver fibrosis and necroinflammatory activity of each group were examined according to the METAVIR scoring system^62^. The METAVIR scoring system consists of five stages, based on the architectural features of portal fibrosis: F0 = normal, F1 = portal fibrosis without septa, F2 = portal fibrosis and few septa, F3 = numerous septa without cirrhosis, and F4 = cirrhosis^63,64^.

### Liver tissue mitochondria and mtDNA extraction

Mitochondria were isolated from liver tissue samples of 14 BA patients and five CC patients using a mitochondrial isolation kit for tissue (Thermo Scientific, Rockford, IL). For the purpose of this analysis, option A (Isolation of Mitochondria from Soft Tissues) from the manufacturer’s protocol 1: Reagent-based Method for Soft Tissues was used. Briefly, each liver tissue (11 ~ 80 mg) resuspended in 800 µL PBS were initially disrupted and homogenized using a pre-chilled 2 ml glass dounce (Wheaton, Millville, NY) to purify mitochondria.

Mitochondrial DNAs (mtDNAs) were extracted from the isolated mitochondrial samples using a QIAamp DNA Mini Kit (Quiagen, Valencia, CA) as per option ‘Isolation of genomic DNA from bacterial cultures’. The extracted mitochondrial DNAs were quantified by Qubit 2.0 fluorometer using a Qubit dsDNA HS assay Kit (Life Technologies, Carlsbad, CA) and qualified by 1% agarose gel electrophoresis.

### Determination of mtDNA Copy Number

Total DNA was extracted from liver samples. The mtDNA copy numbers were measured by real-time PCR using a modified method of Tiao *et al*.^65^, as previously described^66^. The PCR primers complementary to nuclear β-actin gene were 5′-GAAATCGTGCGTGACATTAAAG-3′ and 5′-ATCGGAACCGCTCATTG-3′. The primers to detect mtDNA were complementary to the mitochondrial COX1 gene, which were 5′-TTCGCCGACCGTTGACTATTCTCT-3′ and 5′-AAGATTATTACAAATGCATGGGC-3′. PCR was performed in a BioRad CFX96 real-time PCR detection system (Bio-Rad, Hercules, CA) using the iQ SYBR Green Supermix kit. DNA (10 pg) was mixed with 10 μl of SYBR Green mix containing 10 nmol of primers, in a final volume of 20 μl. The PCR conditions were as follows: initial 50 °C for 2 min, 95 °C for 1 min, 40 cycles of denaturation at 95 °C for 15 s, annealing at 60 °C for 20 s, and primer extension at 72 °C for 15 s. The threshold cycle number (Ct) values of the β-actin gene and the mitochondrial COX1 gene were determined for each individual quantitative PCR run. Ct value differences were used to quantify mtDNA copy number relative to the β-actin gene according to the following equation: the relative copy number (Rc) = 2 ΔCt, where ΔCt is Ct_β-actin_  − Ct_mtDNA_^66^. Each measurement was performed at least 3 times and was normalized against a serial dilution of a control DNA sample.

### Sequencing library construction

Two types of sequencing libraries were prepared as described below. Briefly, direct sequencing library was prepared using total extracted mtDNAs and the mtDNA amplicon library was made by PCR with amplified fragments of mitochondrial DNAs to avoid the nuclear DNA (nDNA) contamination in subsequent making library. For the mtDNA amplicon library, the entire mtDNA was initially amplified as four fragments by PCR using a set of primers specific for mitochondrial origin co-amplification to prevent nDNA sequences^60^. As shown in Fig. 1b, mtDNA fragments 1 to 4 were amplified from mtDNA using the primers 14898 F (5′-TAGCCATGCACTACTCACCAGA-3′) and 1677R (5′-GTTTAGCTCAGAGCGGTCAAGT-3′), 1404 F (5′-ACTTAAGGGTCGAAGGTGGATT-3′) and 6739 R (5′-GATATCATAGCTCAGACCATACC-3′), 6511 F (5′-CTGCTGGCATCACTATACTACTA-3′) and 10648 R (5′-GGCACAATATTGGCTAAGAGGG-3′), and 10360 F (5′-GTCTGGCCTATGAGTGACTACA-3′) and 15349 R (5′-GTGCAAGAATAGGAGGTGGAGT-3′), respectively. The PCR mixture (50 μl) contained 2.5 mM MgCl_2_, 20 ng of mtDNA, 10 pmol of each primer, 0.2 mM dNTP mix, and 1.25 U of PrimeSTAR HS DNA polymerase (Takara Bio, Ohtsu, Japan). After the initial denaturation for 2 min at 95 °C, the DNA was amplified during 30 cycles of 20 sec denaturation at 95 °C, 40 sec annealing at 57 °C and 5 min extension at 72 °C; this was followed by a final extension step of 5 min at 72 °C. The size of PCR products were confirmed by 1% agarose gel electrophoresis, purified further using an AMPure kit (Beckman Coulter, Brea, CA) and pooled in equimolar amounts to generate fragment-sequencing libraries. For the 200 bp libraries, 30~300 ng mtDNA or entire amplified DNA was sheared by Bioruptor Sonication system (Diagenode, Denville, NJ). The rest of steps in the library preparation were performed using the Ion Plus Fragment Library Kit and the Ion DNA Barcoding 1–16 Kit (Life Technologies, Gaithersburg, MD) according to the manufacturer’s instructions. After the sheared DNA end-repair and adapter ligation steps, the library was made from mtDNA size-selected using a 2% E-Gel SizeSelect (Invitrogen, Carlsbad, CA) instrument, followed by 8 cycles of final PCR amplification. The quantification and size distribution of each library was performed by Bioanalyzer 2100 (Agilent Technologies, Santa Clara, CA).

### Sequencing on an ion torrent platform

For the clonal amplified sequencing templates, the libraries were pooled in equimolar amounts and emulsion PCR on Ion OneTouch system with Ion OneTouch 200 Template kit v2 (Life Technologies, Gaithersburg, MD). The templates were automatically enriched with the Ion OneTouch ES system (Life Technologies). Next Generation Sequencing was performed using the Ion Torrent Personal Genome Machine (PGM) sequencer system using a 316D sequencing chip (Life Technologies)^67^. Raw sequence data were analyzed by Ion Torrent Suite v4.0.2.

### Sequence analysis of the mitochondrial genome

*Homo sapiens* mitochondrial complete genome (NC_012920.1) was used as the reference sequence. FASTQ format sequence files were applied to sequence read mapping and variant calling by using the “map read to reference” tool and a “quality-based variant detection” tool in the CLC genomics workbench v7.0.3 software (CLC-bio, Aarhus, Denmark). The “Map read to reference” tool was used with default parameters applying bioinformatic costs for mismatches of “2”, indel costs of “3”, length fraction of “0.5”, similarity fraction of “0.8” was performed. Quality-based variant detection with default parameters neighborhood radius of “5”, maximum gap and mismatch count of “2”, minimum neighborhood quality of “15”, minimum central quality of “20”, minimum coverage of “10”, minimum variant frequency (%) of “35”, variant filter of “Ion homopolymer indels” and genetic code “2 vertebrate mitochondrial”. The mitochondrial DNA haplogroups and mutations in encoding regions were determined by using MITOMAP^68^ and MitoTool^69^.

### Structural mapping and analysis

Thirty-four mutation sites in mtDNA protein coding genes were mapped onto their corresponding human model structures. For human complexes I to V models, the SWISS-MODEL server was used with available high-resolution structures of the human, bovine, ovine and bacterial enzymes (PDB accession codes, 4HEA (for subunits ND1, ND2, ND4L, and ND6), 5XTC (ND3), 5LNK (ND5), 1LOL (Cyt *b*), 3ABM (COX1, COX2, and COX3), and 5ARA (ATP6)) as template structures.

For mutational effect on the structure and function of the respiratory chain complexes, predictive approaches for amino acid substitutions (AAS) analysis were performed using SIFT (http://sift.jcvi.org), PROVEAN (http://provean.jcvi.org), and Polyphen-2 (http://genetics.bwh.harvard.edu/pph/) web servers.

### Multivariate statistical analysis

The redundancy analysis (RDA) was done by using the rda function in the vegan library of the R software version 3.4.1 (www.r-project.org)^70,71^. Instead of relative abundance data, we used measures of heteroplasmic sites including SNPs and InDels, and the histological and clinical criteria in our analysis is defined by phenotype variables. The statistical significance of phenotype variables was assessed with the function anova.cca in vegan package, using 999 permutations to test for the marginal significance of each term after accounting for the effects of the others. The permutation method was implemented to assess whether certain genomic regions (functional genes) were enriched for phenotype-associated variations by calculating empirical P-values of the phenotypes variables with respect to variantation matrix.

## Electronic supplementary material

Supplementary information

Supplementary Table S3
