# Supplementary material for: Mitochondrial Mutations in Cholestatic Liver Disease with Biliary Atresia
Source: Sci Rep. 2018 Jan 17;8:905. doi: 10.1038/s41598-017-18958-8 (PMC5772057; doi:10.1038/s41598-017-18958-8)
Supplement: Supplementary file 1 — Supplementary information [file 41598_2017_18958_MOESM1_ESM.pdf]

Supplementary Information for the paper entitled:

Mitochondrial Mutations in Cholestatic Liver Disease with Biliary Atresia.

Hong Koh§[1], Gun-Seok Park§[2,4], Sun-Mi Shin[2,5], Chang Eon Park[2], Seung Kim[1], Seok Joo Han[3], Huy Quang Pham[2], Jae-Ho Shin[2*], and Dong-Woo Lee[2*]

[1]Department of Pediatrics, Yonsei University College of Medicine, Severance Children's Hospital, Severance Pediatric Liver Research Group, Seoul 03722, South Korea.

[2]School of Applied Biosciences, Kyungpook National University, Daegu 41566, South Korea.

[3]Department of Pediatric Surgery, Yonsei University College of Medicine, Severance Children's Hospital, Seoul 03722, South Korea.

[4]Current address: Department of Biomedical Engineering, University of Texas at Austin, TX 78712, USA.

[5]Current address: CJ CheilJedang, Food Research Institute, Suwon 16495, South Korea.

<Supplementary Tables and Figures>

Table S1. Single nucleotide variations (SNVs) in total DNA direct sequencing and mitochondrial DNA amplicon sequencing

Table S2. Pattern analysis of mitochondrial DNA

Table S3. Total single nucleotide variations (SNVs) in mitochondrial DNA amplicon sequencing (See the MS Excel file saved as "Table S3 SNV locus.xlsx").

Figure S1. Sequence alignment of mitochondrial-encoded ND1 subunits within the complex I between human and its homologs. Amino acid changes are colored in red with bold-face. Human_ND1, *Homo sapiens* ND1 (NCBI protein accession number. YP_003024026.1);

bovine_ND1, *Bos taurus* ND1 (ADF49509.1). T. th_NQO8, *Thermus aquaticus thermophilus* NQO8 (AAA97945.1).

Figure S2. Sequence alignment of mitochondrial-encoded ND2 subunits within the complex I between human and its homologs. Amino acid changes are colored in red with bold-face. Human_ND2, *Homo sapiens* ND2 (YP_003024027.1); bovine_ND2, *Bos taurus* ND2 (ADF49510.1). T. th_NQO14, *T. thermophilus* NQO14 (AAA97951.1).

Figure S3. Sequence alignment of mitochondrial-encoded ND3 subunits within the complex I between human and its homologs. Amino acid changes are colored in red with bold-face. Human_ND3, *Homo sapiens* ND3 (YP_003024033.1); bovine_ND3, *Bos taurus* ND3 (ADF49516.1). T. th_NQO7, *T. thermophilus* NQO7 (AAA97938.1).

Figure S4. Sequence alignment of mitochondrial-encoded ND4 subunits within the complex I between human and its homologs. Amino acid changes are colored in red with bold-face. Human_ND4, *Homo sapiens* ND4 (YP_003024035.1); bovine_ND4, *Bos taurus* ND4 (ADF49518.1). T. th_NQO13, *T. thermophilus* NQO13 (AAA97950.1).

Figure S5. Sequence alignment of mitochondrial-encoded ND5 subunits within the complex I between human and its homologs. Amino acid changes are colored in red with bold-face. Human_ND5, *Homo sapiens* ND5 (YP_003024036.1); bovine_ND5, *Bos taurus* ND5 (ADF49519.1). T. th_NQO12, *T. thermophilus* NQO12 (AAA97949.1).

Figure S6. Sequence alignment of mitochondrial-encoded Cyt *b* subunits within the complex III between human and its homolog. Amino acid changes are colored in red with bold-face. Human_Cytb, *Homo sapiens* cytochrome *b* (YP_003024038.1); bovine_Cytb, *Bos taurus* cytochrome *b* (ADF49521.1).

Figure S7. Sequence alignment of mitochondrial-encoded COX1 subunits within the complex IV between human and its homolog. Amino acid changes are colored in red with bold-face. Human_COX1, *Homo sapiens* cytochrome *c* oxidase subunit I (YP_003024028.1);

bovine_COX1, *Bos taurus* cytochrome *c* oxidase subunit I (ADF49511.1).

Figure S8. Sequence alignment of mitochondrial-encoded COX2 subunits within the complex IV between human and its homolog. Amino acid changes are colored in red with bold-face. Human_COX2, *Homo sapiens* cytochrome *c* oxidase subunit II (YP_003024029.1); bovine_COX2, *Bos taurus* cytochrome *c* oxidase subunit II (ADF49512.1).

Figure S9. Sequence alignment of mitochondrial-encoded COX3 subunits within the complex IV between human and its homolog. Amino acid changes are colored in red with bold-face. Human_COX3, *Homo sapiens* cytochrome *c* oxidase subunit III (YP_003024032.1); bovine_COX3, *Bos taurus* cytochrome *c* oxidase subunit III (ADF49515.1).

Figure S10. Sequence alignment of mitochondrial-encoded ATP6 subunits within the complex V between human and its homolog. Amino acid changes are colored in red with bold-face. Human_ATP6, *Homo sapiens* ATP synthase F0 subunit 6 (YP_003024031.1); bovine_ATP6, *Bos taurus* ATP synthase F0 subunit 6 (ADF49514.1).

Figure S11. Sequence alignment of mitochondrial-encoded ATP8 subunits within the complex V between human and its homolog. Amino acid changes are colored in red with bold-face. Human_ATP8, *Homo sapiens* ATP synthase F0 subunit 8 (YP_003024030.1); bovine_ATP8, *Bos taurus* ATP synthase F0 subunit 8 (ADF49513.1).

*To whom correspondence should be addressed:

Jae-Ho Shin, School of Applied Biosciences, Kyungpook National University, Daegu 41566, South Korea. Tel.: +82-53-950-5716; Fax: +82-53-953-7233; E-mail: jhshin@knu.ac.kr

Dong-Woo Lee, School of Applied Biosciences, Kyungpook National University, Daegu 41566, South Korea. Tel.: +82-53-950-5718; Fax: +82-53-953-7233.; E-mail: leehicam@knu.ac.kr

[§]These authors equally contributed to this work.

Table S1. Single nucleotide variations (SNVs) in total DNA direct sequencing and mitochondrial DNA amplicon sequencing

Patient					Direct seq.			Ampli seq.		
	**Position**	Type	Reference	Allele	Count	Coverage	Frequency	Count	Coverage	Frequency
12	**73**	SNV	A	G	48	48	100	9570	9593	99.76
	**188**	SNV	A	G	43	43	100	9295	9361	99.29
	**235**	SNV	A	G	38	38	100	6893	6915	99.68
	**263**	SNV	A	G	31	31	100	5688	5699	99.81
	**523~524**	Deletion	A	0	16	18	88.89	2072	2436	85.06
	**663**	SNV	A	G	32	33	96.97	8317	8348	99.63
	**750**	SNV	A	G	37	38	97.37	10132	10336	98.03
	**1438**	SNV	A	G	60	60	100	7681	7698	99.78
	**1736**	SNV	A	G	29	29	100	20	20	100
	**2156**	Insertion	0	A	11	62	17.74			
	**2706**	SNV	A	G	39	39	100	41	41	100
	**3107**	Deletion	N	0	49	51	96.08	54	54	100
	**4248**	SNV	T	C	8	13	61.54	13	17	76.47
	**4655**	SNV	G	A	47	47	100	23	23	100
	**4769**	SNV	A	G	52	54	96.3	29	29	100
	**4824**	SNV	A	G	50	51	98.04	27	29	93.1
	**6915**	SNV	G	A	46	46	100	94	94	100
	**7028**	SNV	C	T	66	66	100	148	148	100
	**8563**	SNV	A	G	37	40	92.5	35	35	100
	**8794**	SNV	C	T	43	44	97.73	86	86	100
	**8860**	SNV	A	G	48	49	97.96	86	86	100
	**11536**	SNV	C	T	34	34	100	309	338	91.42
	**11647**	SNV	C	T	28	38	73.68	359	364	98.63
	**11719**	SNV	G	A	42	42	100	332	338	98.22
	**12705**	SNV	C	T	40	53	75.47	413	550	75.09
	**14757**	SNV	T	C	17	19	89.47	123	128	96.09
	**14766**	SNV	C	T	20	22	90.91	110	114	96.49
	**15326**	SNV	A	G	28	28	100	4686	4734	98.99
	**16187**	SNV	C	T	48	48	100	3886	3973	97.81

	**16209**	SNV	T	C	51	51	100	4271	4282	99.74
	**16223**	SNV	C	T	41	42	97.62	3195	3649	87.56
	**16290**	SNV	C	T	29	29	100	4021	4048	99.33
	**16319**	SNV	G	A	22	22	100	3443	4738	72.67
17	**73**	SNV	A	G	15	15	100	9859	9881	99.78
	**235**	SNV	A	G	13	13	100	7308	7342	99.54
	**263**	SNV	A	G	10	10	100	6160	6180	99.68
	**523~524**	Deletion	A	0	12	12	100			
	**663**	SNV	A	G	20	20	100	8314	8357	99.49
	**750**	SNV	A	G	25	25	100	10037	10251	97.91
	**1438**	SNV	A	G	32	34	94.12	8361	8376	99.82
	**1736**	SNV	A	G	13	13	100	54	56	96.43
	**2156**	Insertion	0	A	16	25	84			
	**2706**	SNV	A	G	25	25	100	121	121	100
	**3107**	Deletion	N	0	28	28	100	150	150	100
	**4248**	SNV	T	C	9	13	69.23	34	41	82.93
	**4655**	SNV	G	A	22	23	95.65	72	74	97.3
	**4736**	SNV	T	C	29	29	100	89	90	98.89
	**4769**	SNV	A	G	27	29	93.1	97	98	98.98
	**4824**	SNV	A	G	19	24	79.17	98	100	98
	**5773**	SNV	G	A	25	25	100	84	84	100
	**7028**	SNV	C	T	34	34	100	1107	1109	99.82
	**8563**	SNV	A	G	13	20	65	259	264	98.11
	**8794**	SNV	C	T	21	21	100	596	599	99.5
	**8860**	SNV	A	G	19	19	100	613	616	99.51
	**10801**	SNV	G	A	23	24	95.83	1981	2003	98.9
	**11536**	SNV	C	T	17	17	100	1307	1427	91.59
	**11647**	SNV	C	T	23	27	85.19	1468	1480	99.19
	**11719**	SNV	G	A	27	29	93.1	1422	1428	99.58
	**12705**	SNV	C	T	15	18	83.33	1565	2013	77.74
	**12880**	SNV	T	C	25	25	100	2092	2098	99.71
	**14766**	SNV	C	T				420	439	95.67
	**14944**	SNV	C	T	19	19	100	11093	11207	98.98

	**15326**	SNV	A	G	24	24	100	5398	5436	99.3
	**16187**	SNV	C	T	13	14	92.86	3861	3978	97.06
	**16223**	SNV	C	T	10	13	76.92	2894	3425	84.5
	**16290**	SNV	C	T	10	10	100	3977	4019	98.95
	**16319**	SNV	G	A	10	11	90.91	3340	4566	73.15
18	**73**	SNV	A	G	19	19	100	4721	4734	99.73
	**263**	SNV	A	G	18	18	100	2970	2974	99.87
	**709**	SNV	G	A	18	18	100	4285	4355	98.39
	**750**	SNV	A	G	18	18	100	4689	4788	97.93
	**1119**	SNV	T	C	28	28	100	3616	3633	99.53
	**1438**	SNV	A	G	24	24	100	5283	5292	99.83
	**2706**	SNV	A	G	31	31	100	748	751	99.6
	**3107**	Deletion	N	0	27	28	96.43	904	909	99.45
	**3497**	SNV	C	T	19	20	95	288	294	97.96
	**4769**	SNV	A	G	30	31	96.77	652	661	98.64
	**7028**	SNV	C	T	36	36	100	1816	1831	99.18
	**7772**	SNV	A	G	34	34	100	1293	1328	97.36
	**8281~8289**	Deletion	CCCCCT CTA	0	27	39	69.23			
	**8860**	SNV	A	G	17	17	100	1110	1110	100
	**10310**	SNV	G	A	39	39	100	1078	1085	99.35
	**11719**	SNV	G	A	31	31	100	1697	1706	99.47
	**14133**	SNV	A	G	17	17	100	886	891	99.44
	**14766**	SNV	C	T	12	12	100	447	461	96.96
	**15326**	SNV	A	G	35	35	100	3726	3754	99.25
	**15346**	SNV	G	A	34	34	100	2435	4010	60.72
	**15924**	SNV	A	G	29	30	96.67	4260	4264	99.91
	**16086**	SNV	T	C	27	27	100	3811	3840	99.24
	**16183**	SNV	A	C				262	431	60.79
	**16189**	SNV	T	C				784	921	85.12
	**16217**	SNV	T	C	12	12	100	1463	1481	98.78
	**16311**	SNV	T	C	17	17	100	2412	2414	99.92
	**16519**	Deletion	T	0	14	21	66.67	3889	4108	94.67

Table S2. Pattern analysis of mitochondrial DNA

Patients	Diagnosis	Haplogroup	SNV row count	Total read count	Mapped mtDNA read count	Average Coverage
1	CC	D4a	41	264,985	244,915	2,389×
2	CC	N9a8	26	121,290	118,291	1,739×
3	CC	N9a3	27	249,601	240,810	4,028×
4	CC	G2a1d1a	46	355,254	345,667	5,119×
5	CC	D4e2	36	425,521	410,639	6,535×
6	BA	D5b1a	42	162,707	150,677	1,490×
7	BA	D5a2a1a	45	302,545	278,928	2,777×
8	BA	D4h1a1	42	146,728	141,966	2,052×
9	BA	A5a1a1b	35	152,570	148,301	2,330×
10	BA	D4	36	101,832	99,088	1,578×
11	BA	B4a2b	32	248,273	230,326	2,264×
12	BA	A5a	33	226,135	210,745	2,108×
13	BA	D4	40	203,118	197,954	2,971×
14	BA	A11	31	99,617	97,557	1,596×
15	BA	M8a3a1	39	76,196	73,945	1,119×
16	BA	B4c1a1	29	577,938	564,294	8,472×
17	BA	A5a1a1b	34	314,756	293,863	2,807×
18	BA	B4c1a1a	27	251,829	232,972	2,250×
19	BA	D5b	45	454,096	441,527	7,318×
**Total**	-	-	678	4,734,991	4,522,465	-

Table S3. Total single nucleotide variations (SNVs) in mitochondrial DNA amplicon sequencing (See the MS Excel file saved as "Table S3 SNV locus.xlsx"

Figure S1. Sequence alignment of mitochondrial-encoded ND1 subunits within the complex I between human and its homologs. Amino acid changes are colored in red with bold-face. Human_ND1, *Homo sapiens* ND1 (NCBI protein accession number. YP_003024026.1); bovine_ND1, *Bos taurus* ND1 (ADF49509.1). T. th_NQO8, *Thermus aquaticus thermophilus* NQO8 (AAA97945.1).

```
                               10        20        30        40        50        60        70
                      ....|....|....|....|....|....|....|....|....|....|....|....|....|....|
human_ND1         1   -----------MPMANLLLLIVPILIAMAFLMLTERKILGYMQLRKGPNVVGPYGLLQPFADAMKLFTKE 59
bovine_ND1        1   -----------MFMINILMLIIPILLAVAFLTLVERKVLGYMQLRKGPNVVGPYGLLQPIADAIKLFIKE 59
T.th_NQO8         1   MTWSYPVDPYWMVALKALLVVVGLLTAFAFMTLIERRLLARFQVRMGPNRVGPFGLLQPLADAIKSIFKE 70
Clustal Consensus 1              *   : *:::: :* *.**: * **::*. :*:* *** ***:*****:***:* : ** 44

                               80        90       100       110       120       130       140
                      ....|....|....|....|....|....|....|....|....|....|....|....|....|....|
human_ND1         60  PLKPATSTITLYITAPTLALTIALLLWTPLPMPNP---------LVNLNLGLLFILATSSLAVYSILWSG 120
bovine_ND1        60  PLRPATSSASMFILAPIMALGLALTMWIPLPMPYP---------LINMNLGVLFMLAMSSLAVYSILWSG 120
T.th_NQO8         71  DIVVAQADRFLFVLAPLISVVFALLAFGLIPFGPPGSFFGYQPWVINLDLGILYLFAVSELAVYGIFLSG 140
Clustal Consensus 44   :  * :   ::: ** ::: :**  :  :*:  *         ::*::**:*:::* *.****.*: ** 85

                              150       160       170       180       190       200       210
                      ....|....|....|....|....|....|....|....|....|....|....|....|....|....|
human_ND1         121 WASNSNYALIGALRAVAQTISYEVTLAIILLSTLLMSGSFNLSTLITTQ-EHLWLLLPSWPLAMMWFIST 189
bovine_ND1        121 WASNSKYALIGALRAVAQTISYEVTLAIILLSVLLMSGSFTLSTLITTQ-EQMWLILPAWPLAMMWFIST 189
T.th_NQO8         141 WASGSKYSLLGSLRSSASLISYELGLGLALLAPVLLVGSLNLNDIVNWQKEHGWLFLYAFPAFLVYLIAS 210
Clustal Consensus 86  ***.*:*:*:*:**: *. ****: *.: **: :*: **:.*. ::. * *: **:* ::*  ::::*:: 136

                              220       230       240       250       260       270       280
                      ....|....|....|....|....|....|....|....|....|....|....|....|....|....|
human_ND1         190 LAETNRTPFDLAEGESELVSGFNIEYAAGPFALFFMAEYTNIIMMNTLTTTIFLGTTYDALSPELYTTYF 259
bovine_ND1        190 LAETNRAPFDLTEGESELVSGFNVEYAAGPFALFFMAEYANIIMMNIFTAILFLGTSHNPHMPELYTINF 259
T.th_NQO8         211 MAEAARTPFDLPEAEQELVGGYHTEYSSIKWALFQMAEYIHFITASALIPTLFLG-GWTMPVLEVPYLWM 279
Clustal Consensus 137 :**: *:****.*.*.***.*:: **::  :*** **** ::*  . : . :***        *:    : 177

                              290       300       310       320       330       340       350
                      ....|....|....|....|....|....|....|....|....|....|....|....|....|....|
human_ND1         260 VTKTLLLTSLFLWIRTAYPRFRYDQLMHLLWKNFLPLTL-----------------------ALLMWYVS 306
bovine_ND1        260 TIKSLLLTMSFLWIRASYPRFRYDQLMHLLWKNFLPLTL-----------------------ALCMWHVS 306
T.th_NQO8         280 FLKIAFFLFFFIWIRATWFRLRYDQLLRFGWGFLFPLALLWFLVTALVVALDLPRTYLLYLSALSFLVLL 349
Clustal Consensus 177   *  ::   *:***::: *:*****::: *  ::**:*                       ** :  :  209

                              360
                      ....|....|....|.
human_ND1         307 MPITISSIPPQT---- 318
bovine_ND1        307 LPILTSGIPPQT---- 318
T.th_NQO8         350 GAVLYTPKPARKGGGA 365
Clustal Consensus 209  .:  :  *.:.     213
```

Figure S2. Sequence alignment of mitochondrial-encoded ND2 subunits within the complex I between human and its homologs. Amino acid changes are colored in red with bold-face. Human_ND2, *Homo sapiens* ND2 (YP_003024027.1); bovine_ND2, *Bos taurus* ND2 (ADF49510.1). T. th_NQO14, *T. thermophilus* NQO14 (AAA97951.1).

```
                               10        20        30        40        50        60        70
                       ....|....|....|....|....|....|....|....|....|....|....|....|....|....|
human_ND2         1    ---------------------------------------------------------------------- 1
bovine_ND2        1    ---------------------------------------------------------------------- 1
T.th_NQO14        1    MTLAILAVFSVALTLLGFVLPPQGVKRATLLGLALALASLLLTWGKPFAFGPYAVDGVSQVFTLLALLGA 70
Clustal Consensus 1                                                                           1

                               80        90       100       110       120       130       140
                       ....|....|....|....|....|....|....|....|....|....|....|....|....|....|
human_ND2         1    --------MNPLAQPVIYSTIFAGTLITALSSHWFFTWVGLEMNMLAFIPVLTKKMNPRSTEAAIKYFLT 62
bovine_ND2        1    --------MNPIIFIIILLTIMLGTIIVMISSHWLLVWIGFEMNMLAIIPIMMKNHNPRATEASTKYFLT 62
T.th_NQO14        71   LWTVGLVRSGGFEFYLLVLYAALGMHLLASTRHLLLMLVALEALSLPLYALATWRRG-QGLEAALKYFLL 139
Clustal Consensus 1             . :   ::      *  :   : * ::  :.:*   *.: .:   . . :. **: ****  23

                              150       160       170       180       190       200       210
                       ....|....|....|....|....|....|....|....|....|....|....|....|....|....|
human_ND2         63   QATASMILLMAILFNNMLSGQWTMTNTTN----QYSSLMIMMAMAMKLGMAPFHFWVPEVTQGTPLTSGL 128
bovine_ND2        63   QSTASMLLMMAVIINLMFSGQWTVMKLFN----PMASMLMTMALAMKLGMAPFHFWVPEVTQGIPLSSGL 128
T.th_NQO14        140  GALAAAFFLYGAALFYGDTGSLVLGAPGEGPLYALALGLLLVGLGFKAALAPFHFWTPDVYQGSPTPVVL 209
Clustal Consensus 23    : *: ::: .  :    :*. .:    :      :  :: :.:.:* .:******.*:* ** * .  * 55

                              220       230       240       250       260       270       280
                       ....|....|....|....|....|....|....|....|....|....|....|....|....|....|
human_ND2         129  LLLTWQKLAPISIMYQISPSLNVSLLLTLSILSIMAGSWGGLNQTQLRKILAYSSITHMGWMMAVLPYNP 198
bovine_ND2        129  ILLTWQKLAPMSVLYQIFPSINLNLILTLSVLSILIGGWGGLNQTQLRKIMAYSSIAHMGWMTAVLPYNP 198
T.th_NQO14        210  FMATSVKAAAFAALLRVAAPP--EAVALLVALSVVVGNLAALAQKEAKRLLAYSSIAHAGYMALALYTGN 277
Clustal Consensus 56   :: *  * *.:: : :: ..   . :  *  **:: *. ..* *.: :::*****:*   *:*  .*  .  90

                              290       300       310       320       330       340       350
                       ....|....|....|....|....|....|....|....|....|....|....|....|....|....|
human_ND2         199  NMTI-LNLTIYIILTTTAFLLLNLNS--STTTLLLSRTWNKLTWLTPLIPSTLLSLGGLPPLTGFLPKWA 265
bovine_ND2        199  TMTL-LNLIIYIIMTSTMFTMFMANS--TTTTLSLSHTWNKTPIMTVLILATLLSMGGLPPLSGFMPKWM 265
T.th_NQO14        278  AQALGFYLLTYVLATGLAFAVLSQISPDRVPLEALRGLYRKDPLLGLAFLVAMLSLLGLPPLAGFWGKYL 347
Clustal Consensus 90     :: : *  *:: *   * ::   *   ..   *   :.* . :   :  ::**: *****:**  *:  122

                              360       370       380       390       400       410       420
                       ....|....|....|....|....|....|....|....|....|....|....|....|....|....|
human_ND2         266  IIEEFTKNNSLIIPTIMATITLLNLYFYLRLIYSTSITLLPMSNNVKMKWQFEHTKPTPFLPTLIALT-T 334
bovine_ND2        266  IIQEMTKNNSIILPTFMAITALLNLYFYMRLTYSTTLTMFPSTNNMKMKWQFPLMKKMTFLPTMVVLS-T 334
T.th_NQO14        348  AFAEAARAGAWGVLVLALVTSAVSAYYYLGLGLAVFARPEETPFRPGPPWARAAVVAAGSSSSPWGSSPA 417
Clustal Consensus 122   : * :: .:  : .:    : :. *:*: *  :.       . .    *           .:    : : 141

                              430
                       ....|....|....
human_ND2         335  LLLPISPFMLMIL- 347
bovine_ND2        335  MMLPLTPMLSVLE- 347
T.th_NQO14        418  SSSPPWPRGVKITP 431
Clustal Consensus 141     *  *    :    144
```

Figure S3. Sequence alignment of mitochondrial-encoded ND3 subunits within the complex I between human and its homologs. Amino acid changes are colored in red with bold-face. Human_ND3, *Homo sapiens* ND3 (YP_003024033.1); bovine_ND3, *Bos taurus* ND3 (ADF49516.1). T. th_NQO7, *T. thermophilus* NQO7 (AAA97938.1).

```
                               10        20        30        40        50        60        70
                       ....|....|....|....|....|....|....|....|....|....|....|....|....|....|
human_ND3          1   -------MNFALILMINTLLALLLMIITFWLPQLNGYMEKSTPYECGFDPMSPARVPFSMKFFLVAITFL 63
bovine_ND3         1   -------MNLMLALLTNFTLATLLVIIAFWLPQLNVYSEKTSPYECGFDPMGSARLPFSMKFFLVAITFL 63
T.th_NQO7          1   MAPIQEYVGTLIYVGVALFIGVAALLVGALLGPKKPGRAKLMPYESGNDPAGEVKR-FPVHFYVVAMLFI 69
Clustal Consensus  1          :.  : :     :.   :::   *   :    *  ***.* ** . .:  *.::*::**: *: 28

                               80        90       100       110       120
                       ....|....|....|....|....|....|....|....|....|....|..
human_ND3          64  LFDLEIALLLPLPWALQTTNLPLMVMSSLLLIIILALSLAYEWLQKGLDWTE 115
bovine_ND3         64  LFDLEIALLLPLPWASQTANLNTMLTMALFLIILLAVSLAYEWTQKGLEWTE 115
T.th_NQO7          70  LFDVEVAFLWPYAVSAGGLGLYGFLG-VLAFTLLLFVGFLYEWWKGVMRWH- 119
Clustal Consensus  29  ***:*:*:* * . :    .*  ::   * : ::* :.: *** :  : *   55
```

Figure S4. Sequence alignment of mitochondrial-encoded ND4 subunits within the complex I between human and its homologs. Amino acid changes are colored in red with bold-face. Human_ND4, *Homo sapiens* ND4 (YP_003024035.1); bovine_ND4, *Bos taurus* ND4 (ADF49518.1). T. th_NQO13, *T. thermophilus* NQO13 (AAA97950.1).

```
                                10        20        30        40        50        60        70
                       ....|....|....|....|....|....|....|....|....|....|....|....|....|....|
human_ND4          1   -MLKLIVPTIMLLPLTWLSKKHMIWINTTTHSLIISIIPLLFFNQINNNLFSCSPTFSSDPLTTPLLMLT 69
bovine_ND4         1   -MLKYIIPTIMLMPLTWLSKNNMIWVNSTAHSLLISFTSLLLMNQFGDNSLNFSLLFFSDSLSTPLLILT 69
T.th_NQO13         1   MVVLAVLLPVVFGALLLLGLPRALGVLGAGLSFLLNLY-LFLTHPGGVAHAFQAPLLPGAGVYWAFGLDG 69
Clustal Consensus  1    ::  :: .::: .*  *.  . : :  :  *:::.:  *:: :  .      :  : .  :  .: :   26

                                80        90       100       110       120       130       140
                       ....|....|....|....|....|....|....|....|....|....|....|....|....|....|
human_ND4          70  TWLLPLTIMASQRHLSSEPLSRKKLYLSMLISLQISLIMTFTATELIMFYIFFETTLIPTLAIITRWGNQ 139
bovine_ND4         70  MWLLPLMLMASQHHLSKENLTRKKLFITMLISLQLFLIMTFTAMELILFYILFEATLVPTLIIITRWGNQ 139
T.th_NQO13         70  LSALFFLTIALTVFLGALVARVEGRFLGLALLMEGLLLGLFAARDLLVFYVFFEAALIPALLMLYLYGGE 139
Clustal Consensus  26     * :  :*   .*.      :  :: : : ::  *:  *:* :*::**::**::*:*:* ::  :*.: 65

                               150       160       170       180       190       200       210
                       ....|....|....|....|....|....|....|....|....|....|....|....|....|....|
human_ND4          140 PERLNAGTYFLFYTLVGSLPLLIALIYTHNTLGSLNILLLTLTAQELSNSWANNLMWLAYTMAFMVKMPL 209
bovine_ND4         140 TERLNAGLYFLFYTLAGSLPLLVALIYIQNTVGSLNFLMLQYWVQPVHNSWSNVFMWLACMMAFMVKMPL 209
T.th_NQO13         139 -GRTRALYTFVLFTLVGSLPMLAAVLGDRLLSGSPTFLLEDLLAHPLQEEAA-FWVFLGFALAFDIKTPL 207
Clustal Consensus  65    * .*   *:::**.****:* *::  :   ** .:*:    .: : :. :   ::*.  :** :* ** 102

                               220       230       240       250       260       270       280
                       ....|....|....|....|....|....|....|....|....|....|....|....|....|....|
human_ND4          210 YGLHLWLPKAHVEAPIAGSMVLAAVLLKLGGYGMMRLTLILNPLTKHMAY-PFLVLSLWGMIMTSSICLR 278
bovine_ND4         210 YGLHLWLPKAHVEAPIAGSMVLAAVLLKLGGYGMLRITLILNPMTDFMAY-PFIMLSLWGMIMTSSICLR 278
T.th_NQO13         208 FPLHAWLPPFHQENHPSGLADALGTLYKVGVFDFFRFDIPLAPEGFAQAQGLLLFLAALSALYGAWVAFA 277
Clustal Consensus  103 : ** ***  * *   :*     ..* *:* :.::*: : * *     *   ::.*:  . :  : :.:  133

                               290       300       310       320       330       340       350
                       ....|....|....|....|....|....|....|....|....|....|....|....|....|....|
human_ND4          279 QTDLKSLIAYSSISHMALVVTAILIQTPWSFTGAVILMIAHGLTSSLLFCLANSNYERTHSRIMILSQGL 348
bovine_ND4         279 QTDLKSLIAYSSVSHMALVIVAILIQTPWSYMGATALMIAHGLTSSMLFCLANSNYERIHSRTMILARGL 348
T.th_NQO13         278 AKDFKTLLAYAGLSHMGVAALGVFSGTPEGAMGGLYLLAASGVYTGGLFLLAGRLYERTGTLEIGRYRGL 347
Clustal Consensus  133  .*:*:*:**:.:***.:.  .::  ** .  *.  *: * *: :. ** **.  ***  :  :   :** 170

                               360       370       380       390       400       410       420
                       ....|....|....|....|....|....|....|....|....|....|....|....|....|....|
human_ND4          349 QTLLPLMAFWWLLASLANLALPPTINLLGELSVLVTTFSWSNITLLLTGLNMLVTALYSLYMFTTTQWGS 418
bovine_ND4         349 QTLLPLMATWWLLASLTNLALPPTINLIGELFVVMSTFSWSNITIILMGVNMVITALYSLYMLIMTQRGK 418
T.th_NQO13         348 AQSAPGLAALALILFLAMVGLPGLSGFPGEFLTLLGAYKASPWLAALAFLSVIASAAYALTAFQKTFWEE 417
Clustal Consensus  170     * :*   *:  *: :.**   .: **: .:: ::. *     *  :.:: :* *:*  :  *   . 200

                               430       440       450       460       470
                       ....|....|....|....|....|....|....|....|....|....|
human_ND4          419 LTHHINNMKPSFTRENTLMFMHLSPILLLSLNPDIITGFSS-------------- 459
bovine_ND4         419 YTYHINNISPSFTRENALMSLHILPLLLLTLNPKIILGPLY-------------- 459
T.th_NQO13         418 GGSGVKDLAG---AEWGFALLSVLALLLMGVFPGYFARGLHPLAEAFAKLLGGGA 469
Clustal Consensus  200     ::::      *  :  : : .:**: : *  :                    215
```

Figure S5. Sequence alignment of mitochondrial-encoded ND5 subunits within the complex I between human and its homologs. Amino acid changes are colored in red with bold-face. Human_ND5, *Homo sapiens* ND5 (YP_003024036.1); bovine_ND5, *Bos taurus* ND5 (ADF49519.1). T. th_NQO12, *T. thermophilus* NQO12 (AAA97949.1).

```
                               10        20        30        40        50        60        70
                        ....|....|....|....|....|....|....|....|....|....|....|....|....|....|
human_ND5           1   MTMHTTMTTLTLTSLIPPILTTLVNPNKKNSYPHYVKSIVASTFIISLFPTTMFMCLDQEVIISNWHWAT 70
bovine_ND5          1   MNMFSSLSLVTLLLLTMPIMMMNFNTYKPSNYPLYVKTAISYAFITSMIPTMMFIHSGQELIISNWHWLT 70
T.th_NQO12          1   ------------MALLGTILLPLLGFALLGLFGKRMREPLPGVLASGLVLASFLLGAG-LLLSGGARFQA 57
Clustal Consensus   1                 *  .*:   ..    . :   ::  :. .:  .:. : :::  .  :: .. :: : 18

                                 80        90       100       110       120       130       140
                        ....|....|....|....|....|....|....|....|....|....|....|....|....|....|
human_ND5           71  TQTTQLSLSFKLDYFSMMFIPVALFVTWSIMEFSLWYMNSDPNINQFFKYLLIFLITMLILVTANNLFQL 140
bovine_ND5          71  IQTLKLSLSFKMDYFSMMFIPVALFVTWSIMEFSMWYMYSDPNINKFFKYLLLFLITMLILVTANNLFQL 140
T.th_NQO12          58  EWLPGIPFSLLLDNLSGFMLLIVTGVGFLIHVYAIGYMGGDPGYSRFFAYFNLFIAMMLTLVLADSYPVM 127
Clustal Consensus   18       :.:*: :* :* ::: :.  * : *  ::: ** .**. .:** *: :*:  ** ** *:.   : 55

                                150       160       170       180       190       200       210
                        ....|....|....|....|....|....|....|....|....|....|....|....|....|....|
human_ND5           141 FIGWEGVGIMSFLLISWWYARADANTAAIQAILYNRIGDIGFILALAWFILHSNSWDPQQMALLNANPSL 210
bovine_ND5          141 FIGWEGVGIMSFLLIGWWYGRADANTAALQAILYNRIGDIGFILAMAWFLTNLNTWDLQQIFMLNPSDSN 210
T.th_NQO12          128 FIGWEGVGLASFLLIGFWYKNPQYADSARKAFIVNRIGDLGFMLGMAILWALYGTLSISELKEAMEGPLK 197
Clustal Consensus   56  ********: *****.:** ..:   :* :*:: *****:**:*.:* :    .: . .::     .    95

                                220       230       240       250       260       270       280
                        ....|....|....|....|....|....|....|....|....|....|....|....|....|....|
human_ND5           211 TP------LLGLLLAAAGKSAQLGLHPWLPSAMEGPTPVSALLHSSTMVVAGIFLLIRFHPLAENSPLIQ 274
bovine_ND5          211 MP------LIGLALAATGKSAQFGLHPWLPSAMEGPTPVSALLHSSTMVVAGIFLLIRFYPLTENNKYIQ 274
T.th_NQO12          198 NPDLLALAGLLLFLGAVGKSAQIPLMVWLPDAMAGPTPVSALIHAATMVTAGVYLIARSSFLYSVLPDVS 267
Clustal Consensus   95   *       : * *.*.*****: *  ***.** ********:*::***.**::*: *   * .    :. 136

                                290       300       310       320       330       340       350
                        ....|....|....|....|....|....|....|....|....|....|....|....|....|....|
human_ND5           275 TLTLCLGAITTLFAAVCALTQNDIKKIVAFSTSSQLGLMMVTIGINQPHLAFLHICTHAFFKAMLFMCSG 344
bovine_ND5          275 SITLCLGAITTLFTAMCALTQNDIKKIIAFSTSSQLGLMMVTIGINQPYLAFLHICTHAFFKAMLFMCSG 344
T.th_NQO12          268 YAIAVVGLLTAAYGALSAFGQTDIKKIVAYSTISQLGYMFLAAGVGAYWVALFHVFTHAFFKALLFLASG 337
Clustal Consensus   136      :* :*: : *:.*: *.*****:*:** **** *::: *:.   :*::*: *******:**:.** 186

                                360       370       380       390       400       410       420
                        ....|....|....|....|....|....|....|....|....|....|....|....|....|....|
human_ND5           345 SIIHNLNNEQDIRKMGGLLKTMPLTSTSLTIGSLALAGMPFLTGFYSKDHIIETANMS-YTNAWALSITL 413
bovine_ND5          345 SIIHSLNDEQDIRKMGGLFKAMPFTTTALIVGSLALTGMPFLTGFYSKDLIIEAANTS-YTNAWALLMTL 413
T.th_NQO12          338 SVIHALGGEQDVRKMGGLWKHLPQTRWHALIGALALGGLPLLSGFWSKDAILAATLTYPFGGVGFYVGAL 407
Clustal Consensus   187 *:** *..***:****** * :* *     :*:*** *:*:*:**:*** *: ::    : ..     :* 230

                                430       440       450       460       470       480       490
                        ....|....|....|....|....|....|....|....|....|....|....|....|....|....|
human_ND5           414 IATSLTSAYSTRMILLTLTGQPRFPTLTNINENNPTLLNPIKRLAAGSLFAGFLITNNISPASPFQTTIP 483
bovine_ND5          414 IATSFTAIYSTRIIFFALLGQPRFPTLVNINENNPLLINSIKRLLIGSLFAGYIISNNIPPTTIPQMTMP 483
T.th_NQO12          408 LVAVLTAMYAMRWFVLVFLGEERGHHHP--HEAPPVMLWPNHLLALGSVLAGYLALPHPLPNVLEPFLKP 475
Clustal Consensus   231 :.: :*: *: * :.:.: *: *       :*  * :: . : *  **::**::   :  *        * 262

                                500       510       520       530       540       550       560
                        ....|....|....|....|....|....|....|....|....|....|....|....|....|....|
human_ND5           484 LYLKLTALAVTFLGLLTALDLNYLTNKLKMKSPLCTFYFSNMLGFYPSITHR-------TIPYLGLLTSQ 546
bovine_ND5          484 YYLKTTALIVTILGFILALEISNMTKNLKYHYPSNAFKFSTLLGYFPTIMHR-------LAPYMNLSMSQ 546
T.th_NQO12          476 ALAEVEAHHLSLGAEWGLIALSAAVALLGLWAGFVFFQRKVFPAWYLAFEAASREAFYVDRAYNALIVNP 545
Clustal Consensus   262    :  *  ::: .    : :.  .  *        *  . : .:: ::            .*  *  .  278
```

Figure S6. Sequence alignment of mitochondrial-encoded Cyt *b* subunits within the complex III between human and its homolog. Amino acid changes are colored in red with bold-face. Human_Cytb, *Homo sapiens* cytochrome *b* (YP_003024038.1); bovine_Cytb, *Bos taurus* cytochrome *b* (ADF49521.1).

```
                               10        20        30        40        50        60        70
                       ....|....|....|....|....|....|....|....|....|....|....|....|....|....|
Human_Cytb          1   MTPMRKTNPLMKLINHSFIDLPTPSNISAWWNFGSLLGACLILQITTGLFLAMHYSPDASTAFSSIAHIT 70
Bovine_Cytb         1   MTNIRKSHPLMKIVNNAFIDLPAPSNISSWWNFGSLLGICLILQILTGLFLAMHYTSDTTTAFSSVTHIC 70
Clustal Consensus   1   ** :**::****::*::*****:*****:********* ****** *********:.*::*****::**  65

                               80        90       100       110       120       130       140
                       ....|....|....|....|....|....|....|....|....|....|....|....|....|....|
Human_Cytb          71  RDVNYGWIIRYLHANGASMFFICLFLHIGRGLYYGSFLYSETWNIGIILLLATMATAFMGYVLPWGQMSF 140
Bovine_Cytb         71  RDVNYGWIIRYMHANGASMFFICLYMHVGRGLYYGSYTFLETWNIGVILLLTVMATAFMGYVLPWGQMSF 140
Clustal Consensus   66  ***********:************::*:********: : ******:****:.***************** 132

                              150       160       170       180       190       200       210
                       ....|....|....|....|....|....|....|....|....|....|....|....|....|....|
Human_Cytb          141 WGATVITNLLSAIPYIGTDLVQWIWGGYSVDSPTLTRFFTFHFILPFIIAALATLHLLFLHETGSNNPLG 210
Bovine_Cytb         141 WGATVITNLLSAIPYIGTNLVEWIWGGFSVDKATLTRFFAFHFILPFIIMAIAMVHLLFLHETGSNNPTG 210
Clustal Consensus   133 ******************:**:*****:***..******:********* *:* :************* * 197

                              220       230       240       250       260       270       280
                       ....|....|....|....|....|....|....|....|....|....|....|....|....|....|
Human_Cytb          211 ITSHSDKITFHPYYTIKDALGLLLFLLSLMTLTLFSPDLLGDPDNYTLANPLNTPPHIKPEWYFLFAYTI 280
Bovine_Cytb         211 ISSDVDKIPFHPYYTIKDILGALLLILALMLLVLFAPDLLGDPDNYTPANPLNTPPHIKPEWYFLFAYAI 280
Clustal Consensus   198 *:*. ***.********* ** **::*:** *.**:*********** ********************:* 259

                              290       300       310       320       330       340       350
                       ....|....|....|....|....|....|....|....|....|....|....|....|....|....|
Human_Cytb          281 LRSVPNKLGGVLALLLSILILAMIPILHMSKQQSMMFRPLSQSLYWLLAADLLILTWIGGQPVSYPFTII 350
Bovine_Cytb         281 LRSIPNKLGGVLALAFSILILALIPLLHTSKQRSMMFRPLSQCLFWALVADLLTLTWIGGQPVEHPYITI 350
Clustal Consensus   260 ***:********** :******:**:** ***:*********.*:* *.**** *********.:*:  * 320

                              360       370       380
                       ....|....|....|....|....|....|
Human_Cytb          351 GQVASVLYFTTILILMPTISLIENKMLKWA 380
Bovine_Cytb         351 GQLASVLYFLLILVLMPTAGTIENKLLKW- 379
Clustal Consensus   321 **:******  **:**** . ****:***  344
```

Figure S7. Sequence alignment of mitochondrial-encoded COX1 subunits within the complex IV between human and its homolog. Amino acid changes are colored in red with bold-face. Human_COX1, *Homo sapiens* cytochrome *c* oxidase subunit I (YP_003024028.1); bovine_COX1, *Bos taurus* cytochrome *c* oxidase subunit I (ADF49511.1).

```
                             10        20        30        40        50        60        70
                     ....|....|....|....|....|....|....|....|....|....|....|....|....|....|
Human_COX1        1   MFADRWLFSTNHKDIGTLYLLFGAWAGVLGTALSLLIRAELGQPGNLLGNDHIYNVIVTAHAFVMIFFMV 70
Bovine_COX1       1   MFINRWLFSTNHKDIGTLYLLFGAWAGMVGTALSLLIRAELGQPGTLLGDDQIYNVVVTAHAFVMIFFMV 70
Clustal Consensus 1   ** :***********************::****************.***:*:****:************* 68

                             80        90       100       110       120       130       140
                     ....|....|....|....|....|....|....|....|....|....|....|....|....|....|
Human_COX1        71  MPIMIGGFGNWLVPLMIGAPDMAFPRMNNMSFWLLPPSLLLLLASAMVEAGAGTGWTVYPPLAGNYSHPG 140
Bovine_COX1       71  MPIMIGGFGNWLVPLMIGAPDMAFPRMNNMSFWLLPPSFLLLLASSMVEAGAGTGWTVYPPLAGNLAHAG 140
Clustal Consensus 69  **************************************:******:******************* :*.* 136

                            150       160       170       180       190       200       210
                     ....|....|....|....|....|....|....|....|....|....|....|....|....|....|
Human_COX1        141 ASVDLTIFSLHLAGVSSILGAINFITTIINMKPPAMTQYQTPLFVWSVLITAVLLLLSLPVLAAGITMLL 210
Bovine_COX1       141 ASVDLTIFSLHLAGVSSILGAINFITTIINMKPPAMSQYQTPLFVWSVMITAVLLLLSLPVLAAGITMLL 210
Clustal Consensus 137 ************************************:***********:********************* 206

                            220       230       240       250       260       270       280
                     ....|....|....|....|....|....|....|....|....|....|....|....|....|....|
Human_COX1        211 TDRNLNTTFFDPAGGGDPILYQHLFWFFGHPEVYILILPGFGMISHIVTYYSGKKEPFGYMGMVWAMMSI 280
Bovine_COX1       211 TDRNLNTTFFDPAGGGDPILYQHLFWFFGHPEVYILILPGFGMISHIVTYYSGKKEPFGYMGMVWAMMSI 280
Clustal Consensus 207 ********************************************************************** 276

                            290       300       310       320       330       340       350
                     ....|....|....|....|....|....|....|....|....|....|....|....|....|....|
Human_COX1        281 GFLGFIVWAHHMFTVGMDVDTRAYFTSATMIIAIPTGVKVFSWLATLHGSNMKWSAAVLWALGFIFLFTV 350
Bovine_COX1       281 GFLGFIVWAHHMFTVGMDVDTRAYFTSATMIIAIPTGVKVFSWLATLHGGNIKWSPAMMWALGFIFLFTV 350
Clustal Consensus 277 *************************************************.*:***.*::*********** 344

                            360       370       380       390       400       410       420
                     ....|....|....|....|....|....|....|....|....|....|....|....|....|....|
Human_COX1        351 GGLTGIVLANSSLDIVLHDTYYVVAHFHYVLSMGAVFAIMGGFIHWFPLFSGYTLDQTYAKIHFTIMFIG 420
Bovine_COX1       351 GGLTGIVLANSSLDIVLHDTYYVVAHFHYVLSMGAVFAIMGGFVHWFPLFSGYTLNDTWAKIHFAIMFVG 420
Clustal Consensus 345 *******************************************:***********::*:*****:***:* 414

                            430       440       450       460       470       480       490
                     ....|....|....|....|....|....|....|....|....|....|....|....|....|....|
Human_COX1        421 VNLTFFPQHFLGLSGMPRRYSDYPDAYTTWNILSSVGSFISLTAVMLMIFMIWEAFASKRKVLMVEEPSM 490
Bovine_COX1       421 VNMTFFPQHFLGLSGMPRRYSDYPDAYTMWNTISSMGSFISLTAVMLMVFIIWEAFASKREVLTVDLTTT 490
Clustal Consensus 415 **:************************* ** :**:************:*:*********:** *: .:  478

                            500       510
                     ....|....|....|....|....
Human_COX1        491 NLEWLYGCPPPYHTFEEPVYMKS- 513
Bovine_COX1       491 NLEWLNGCPPPYHTFEEPTYVNLK 514
Clustal Consensus 479 ***** ************.*::    498
```

Figure S8. Sequence alignment of mitochondrial-encoded COX2 subunits within the complex IV between human and its homolog. Amino acid changes are colored in red with bold-face. Human_COX2, *Homo sapiens* cytochrome *c* oxidase subunit II (YP_003024029.1); bovine_COX2, *Bos taurus* cytochrome *c* oxidase subunit II (ADF49512.1).

```
                              10        20        30        40        50        60        70
                      ....|....|....|....|....|....|....|....|....|....|....|....|....|....|
Human_COX2        1   MAHAAQVGLQDATSPIMEELITFHDHALMIIFLICFLVLYALFLTLTTKLTNTNISDAQEMETVWTILPA 70
Bovine_COX2       1   MAYPMQLGFQDATSPIMEELLHFHDHTLMIVFLISSLVLYIISLMLTTKLTHTSTMDAQEVETIWTILPA 70
Clustal Consensus 1   **:. *:*:***********: ****:***:***. **** : * ******:*.  ****:**:****** 59

                              80        90       100       110       120       130       140
                      ....|....|....|....|....|....|....|....|....|....|....|....|....|....|
Human_COX2        71  IILVLIALPSLRILYMTDEVNDPSLTIKSIGHQWYWTYEYTDYGGLIFNSYMLPPLFLEPGDLRLLDVDN 140
Bovine_COX2       71  IILILIALPSLRILYMMDEINNPSLTVKTMGHQWYWSYEYTDYEDLSFDSYMIPTSELKPGELRLLEVDN 140
Clustal Consensus 60  ***:************ **:*:****:*::******:****** .* *:***:*.  *:**:****:*** 122

                             150       160       170       180       190       200       210
                      ....|....|....|....|....|....|....|....|....|....|....|....|....|....|
Human_COX2        141 RVVLPIEAPIRMMITSQDVLHSWAVPTLGLKTDAIPGRLNQTTFTATRPGVYYGQCSEICGANHSFMPIV 210
Bovine_COX2       141 RVVLPMEMTIRMLVSSEDVLHSWAVPSLGLKTDAIPGRLNQTTLMSSRPGLYYGQCSEICGSNHSFMPIV 210
Clustal Consensus 123 *****:* .***:::*:*********:***************: ::***:**********:******** 189

                             220
                      ....|....|....|..
Human_COX2        211 LELIPLKIFEMGPVFTL 227
Bovine_COX2       211 LELVPLKYFEKWSASML 227
Clustal Consensus 190 ***:*** **  ..  * 199
```

Figure S9. Sequence alignment of mitochondrial-encoded COX3 subunits within the complex IV between human and its homolog. Amino acid changes are colored in red with bold-face. Human_COX3, *Homo sapiens* cytochrome *c* oxidase subunit III (YP_003024032.1); bovine_COX3, *Bos taurus* cytochrome *c* oxidase subunit III (ADF49515.1).

```
                              10        20        30        40        50        60        70
                      ....|....|....|....|....|....|....|....|....|....|....|....|....|....|
Human_COX3        1   MTHQSHAYHMVKPSPWPLTGALSALLMTSGLAMWFHFHSMTLLMLGLLTNTLTMYQWWRDVTRESTYQGH 70
Bovine_COX3       1   MTHQTHAYHMVNPSPWPLTGALSALLMTSGLTMWFHFNSMTLLMIGLTTNMLTMYQWWRDVIRESTFQGH 70
Clustal Consensus 1   ****:******:*******************:*****:******:** ** ********** ****:*** 67

                              80        90       100       110       120       130       140
                      ....|....|....|....|....|....|....|....|....|....|....|....|....|....|
Human_COX3        71  HTPPVQKGLRYGMILFITSEVFFFAGFFWAFYHSSLAPTPQLGGHWPPTGITPLNPLEVPLLNTSVLLAS 140
Bovine_COX3       71  HTPAVQKGLRYGMILFIISEVLFFTGFFWAFYHSSLAPTPELGGCWPPTGIHPLNPLEVPLLNTSVLLAS 140
Clustal Consensus 68  ***.************* ***:**:***************:*** ****** ****************** 133

                             150       160       170       180       190       200       210
                      ....|....|....|....|....|....|....|....|....|....|....|....|....|....|
Human_COX3        141 GVSITWAHHSLMENNRNQMIQALLITILLGLYFTLLQASEYFESPFTISDGIYGSTFFVATGFHGLHVII 210
Bovine_COX3       141 GVSITWAHHSLMEGDRKHMLQALFITITLGVYFTLLQASEYYEAPFTISDGVYGSTFFVATGFHGLHVII 210
Clustal Consensus 134 *************.:*::*:***:*** **:**********:*:*******:****************** 201

                             220       230       240       250       260
                      ....|....|....|....|....|....|....|....|....|....|.
Human_COX3        211 GSTFLTICFIRQLMFHFTSKHHFGFEAAAWYWHFVDVVWLFLYVSIYWWGS 261
Bovine_COX3       211 GSTFLIVCFFRQLKFHFTSNHHFGFEAAAWYWHFVDVVWLFLYVSIYWWG- 260
Clustal Consensus 202 ***** :**:*** *****:******************************  249
```

Figure S10. Sequence alignment of mitochondrial-encoded ATP6 subunits within the complex V between human and its homolog. Amino acid changes are colored in red with bold-face. Human_ATP6, *Homo sapiens* ATP synthase F0 subunit 6 (YP_003024031.1); bovine_ATP6, *Bos taurus* ATP synthase F0 subunit 6 (ADF49514.1).

```
                             10        20        30        40        50        60        70
                     ....|....|....|....|....|....|....|....|....|....|....|....|....|....|
Human_ATP6        1   MNENLFASFIAPTILGLPAAVLIILFPPLLIPTSKYLINNRLITTQQWLIKLTSKQMMTMHNTKGRTWSL 70
Bovine_ATP6       1   MNENLFTSFITPVILGLPLVTLIVLFPSLLFPTSNRLVSNRFVTLQQWMLQLVSKQMMSIHNSKGQTWTL 70
Clustal Consensus 1   ******:***:*.***** ..**:***.**:***: *:.**::* ***:::*.*****::**:**:**:* 61

                             80        90       100       110       120       130       140
                     ....|....|....|....|....|....|....|....|....|....|....|....|....|....|
Human_ATP6        71  MLVSLIIFIATTNLLGLLPHSFTPTTQLSMNLAMAIPLWAGTVIMGFRSKIKNALAHFLPQGTPTPLIPM 140
Bovine_ATP6       71  MLMSLILFIGSTNLLGLLPHSFTPTTQLSMNLGMAIPLWAGAVITGFRNKTKASLAHFLPQGTPTPLIPM 140
Clustal Consensus 62  **:***:**.:**********************.********:** ***.* * :**************** 125

                            150       160       170       180       190       200       210
                     ....|....|....|....|....|....|....|....|....|....|....|....|....|....|
Human_ATP6        141 LVIIETISLLIQPMALAVRLTANITAGHLLMHLIGSATLAMSTINLPSTLIIFTILILLTILEIAVALIQ 210
Bovine_ATP6       141 LVIIETISLFIQPMALAVRLTANITAGHLLIHLIGGATLALMSISTTTALITFTILILLTILEFAVAMIQ 210
Clustal Consensus 126 *********:*******************:****.****: :*. .::** ***********:***:** 189

                            220
                     ....|....|....|.
Human_ATP6        211 AYVFTLLVSLYLHDNT 226
Bovine_ATP6       211 AYVFTLLVSLYLHDNT 226
Clustal Consensus 190 **************** 205
```

Figure S11. Sequence alignment of mitochondrial-encoded ATP8 subunits within the complex V between human and its homolog. Amino acid changes are colored in red with bold-face. Human_ATP8, *Homo sapiens* ATP synthase F0 subunit 8 (YP_003024030.1); bovine_ATP8, *Bos taurus* ATP synthase F0 subunit 8 (ADF49513.1).

```
                              10        20        30        40        50        60
                     ....|....|....|....|....|....|....|....|....|....|....|....|....|...
Human_ATP8        1  MPQLNTTVWPTMITPMLLTLFLITQLKMLNTNYHLPPSPKPMKMKNYNKPWEPKWTKICSLHSLPPQS 68
Bovine_ATP8       1  MPQLDTSTWLTMILSMFLTLFIIFQLKVSKHNFYHNPELTPTKMLKQNTPWETKWTKIY-LPLLLPL- 66
Clustal Consensus 1  ****:*:.* *** .*:****:* ***: : *::  *. .* ** : *.***.*****  *  * *   44
```
